# Circularly permuted variants of two CG-specific prokaryotic DNA methyltransferases

**DOI:** 10.1371/journal.pone.0197232

**Published:** 2018-05-10

**Authors:** Pál Albert, Bence Varga, Nikolett Zsibrita, Antal Kiss

**Affiliations:** 1 Institute of Biochemistry, Biological Research Centre of the Hungarian Academy of Sciences, Szeged, Hungary; 2 Doctoral School in Biology, Faculty of Science and Informatics, University of Szeged, Szeged, Hungary; Universität Stuttgart, GERMANY

## Abstract

The highly similar prokaryotic DNA (cytosine-5) methyltransferases (C5-MTases) M.MpeI and M.SssI share the specificity of eukaryotic C5-MTases (5’-CG), and can be useful research tools in the study of eukaryotic DNA methylation and epigenetic regulation. In an effort to improve the stability and solubility of complementing fragments of the two MTases, genes encoding circularly permuted (CP) variants of M.MpeI and M.SssI were created, and cloned in a plasmid vector downstream of an arabinose-inducible promoter. MTase activity of the CP variants was tested by digestion of the plasmids with methylation-sensitive restriction enzymes. Eleven of the fourteen M.MpeI permutants and six of the seven M.SssI permutants had detectable MTase activity as indicated by the full or partial protection of the plasmid carrying the cpMTase gene. Permutants cp62M.MpeI and cp58M.SssI, in which the new N-termini are located between conserved motifs II and III, had by far the highest activity. The activity of cp62M.MpeI was comparable to the activity of wild-type M.MpeI. Based on the location of the split sites, the permutants possessing MTase activity can be classified in ten types. Although most permutation sites were designed to fall outside of conserved motifs, and the MTase activity of the permutants measured in cell extracts was in most cases substantially lower than that of the wild-type enzyme, the high proportion of circular permutation topologies compatible with MTase activity is remarkable, and is a new evidence for the structural plasticity of C5-MTases. A computer search of the REBASE database identified putative C5-MTases with CP arrangement. Interestingly, all natural circularly permuted C5-MTases appear to represent only one of the ten types of permutation topology created in this work.

## Introduction

DNA methylation plays important roles in several biological phenomena such as restriction-modification in prokaryotes, genomic imprinting, X-chromosome inactivation and silencing of selfish genetic elements in eukaryotes. Biological DNA methylation is catalyzed by DNA methyltransferases (DNA MTase), which transfer a methyl group from the universal methyl donor S-adenosyl-L-methionine (SAM) to an adenine or cytosine in specific sequences. Depending on the methylated base, DNA MTases can be classified in three groups (N6-adenine-, N4-cytosine- and C5-cytosine MTases [1, 2]. N6-adenine and N4-cytosine MTases transfer the methyl group onto the exocyclic amino group of the respective base, whereas DNA (cytosine-5) methyltransferases (C5-MTases) add the methyl group to carbon 5 of the pyrimidine ring [3]. In contrast to prokaryotes, in which all three types DNA methylation are ubiquitous, eukaryotes typically contain C5-methylcytosine [1, 2]. In higher eukaryotes C5-methylcytosines are important epigenetic marks [4], which occur predominantly in CG dinucleotides.

Most C5-MTases consist of a single polypeptide chain. Prokaryotic C5-MTases are built of ~400 amino acids. Eukaryotic C5-MTases are larger enzymes but their C-terminal part shares sequence similarity with the prokaryotic enzymes [5]. The amino acid sequences of C5-MTases contain ten conserved blocks of amino acids that are characteristic to all members of this enzyme family [3, 5, 6]. Motifs I, II, III and X constitute the binding site for the methyl donor SAM, whereas motifs IV, V, VI, VII, VIII take part in the formation of the catalytic pocket. Sequence specific DNA recognition is mediated by the target recognition domain (TRD), which is located in a variable region between motifs VIII and IX [3, 6–10].

Although the vast majority of characterized C5-MTases contain the conserved motifs in the same sequential order (I through X), there are exceptions: in M.BssHII [11],[12] M.Alw26I, M2.Eco31I, M.Esp3I [13] and M2.BsaI [14] and observations by Zhu and Xu, (cited in REBASE [15]) the order of the conserved blocks is circularly permuted (CP) compared to the canonical arrangement. The N-termini of these enzymes are in the variable region, thus motifs IX and X precede motifs I-VIII.

**Fig 1:**
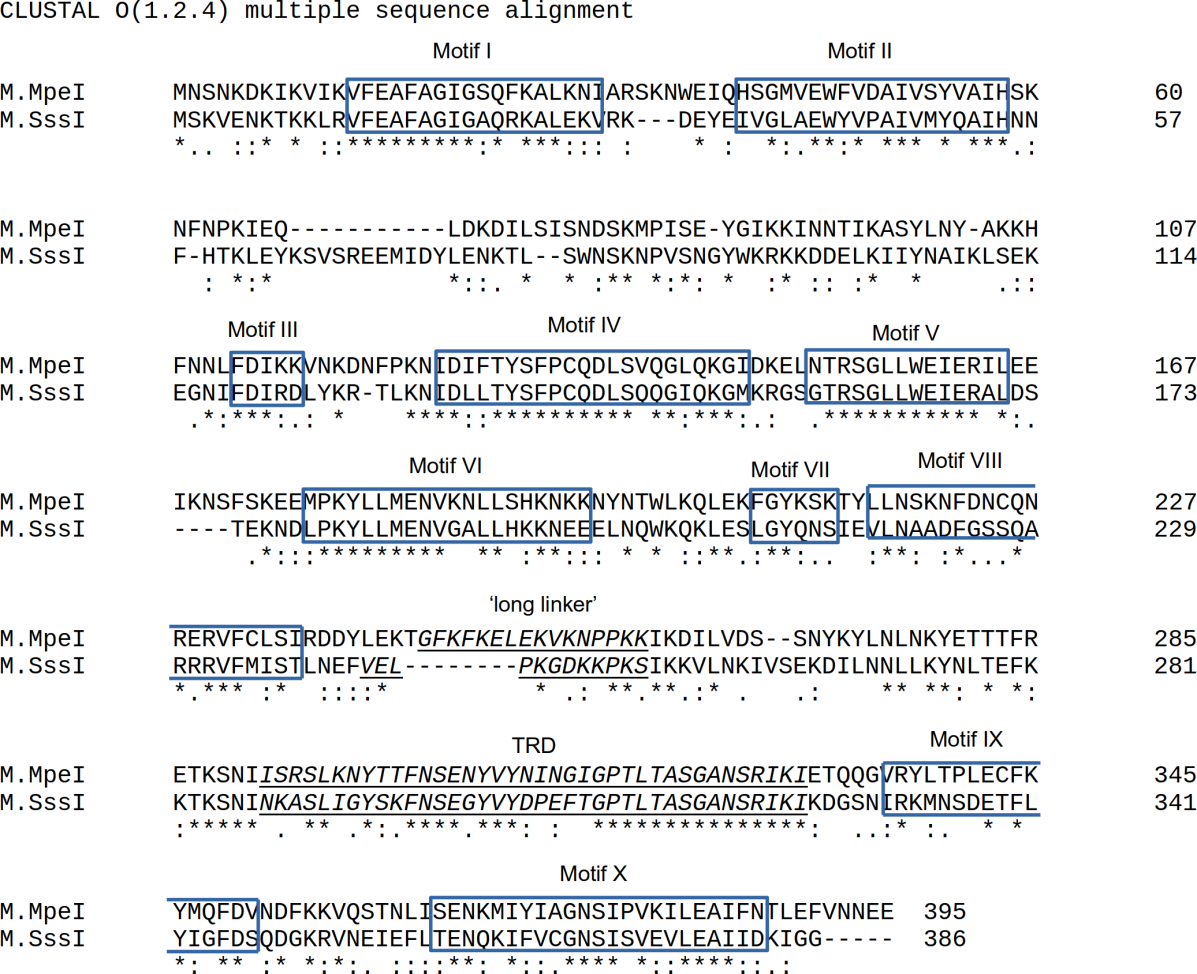
ClustalO sequence alignment between M.MpeI and M.SssI. Identical amino acids are indicated by asterisks, conserved substitutions by colons and semi-conserved substitutions by dots. Conserved motifs are shown according to Koudan *et al*. [23]. The “long linker” and the TRD are marked according to Choe *et al*. [24].

The prokaryotic C5-MTases M.SssI of *Spiroplasma sp*. strain MQ1 [16] and M.MpeI of *Mycoplasma penetrans* [17] have the same sequence specificity (CG) as the mammalian C5-MTases, which makes them valuable tools for studying DNA methylation in higher eukaryotes [18–22]. Both enzymes are monomeric proteins composed of 386 and 395 amino acids, respectively, and have highly similar amino acid sequences (Fig 1).

**Fig 2:**
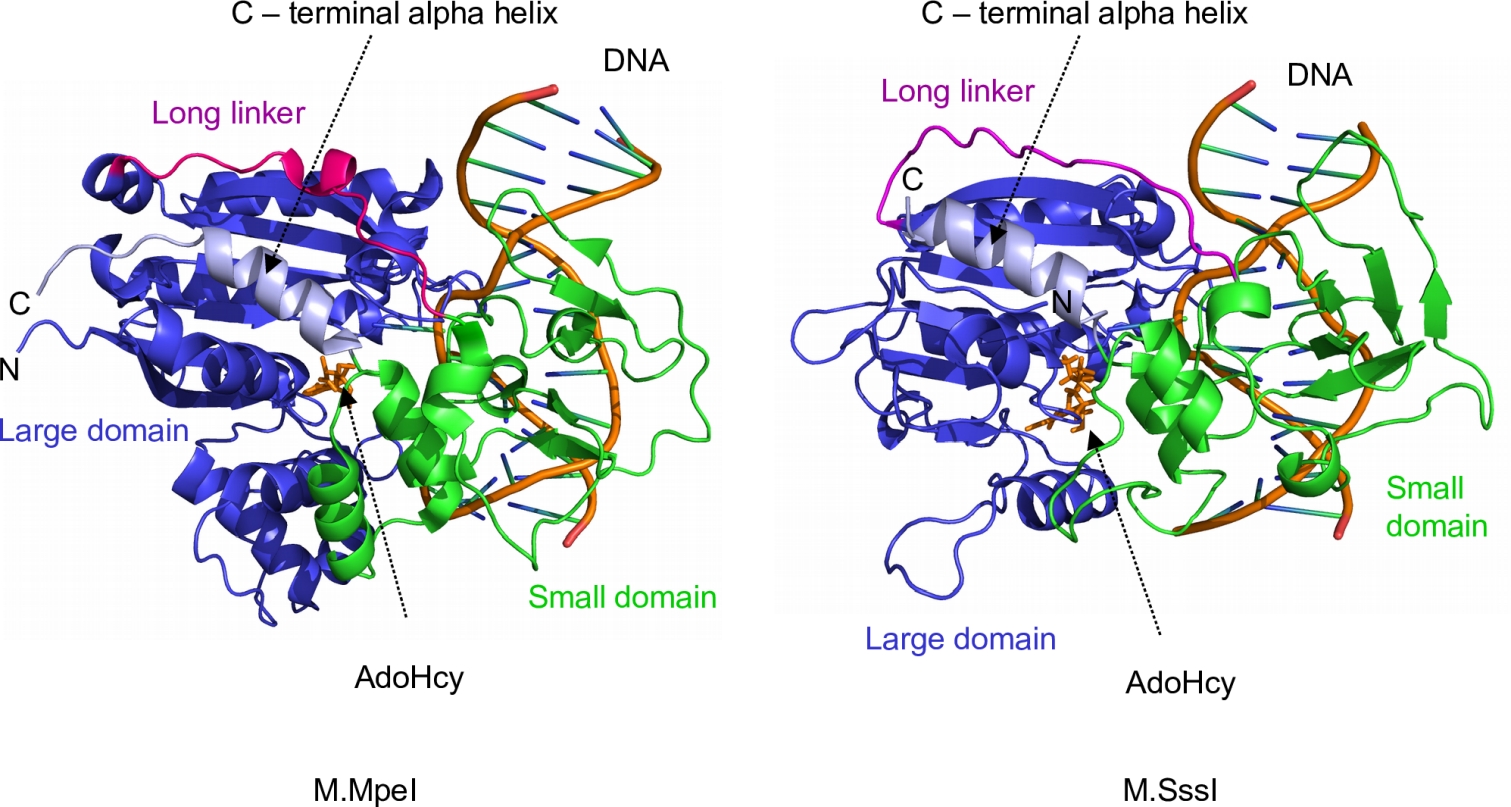
Ternary complexes of the MpeI and SssI DNA methyltransferases. M.MpeI, M.MpeI-DNA-S-adenosyl-homocysteine complex, X-ray crystallographic structure [30] (PDB: 4DKJ). M.SssI, M.SssI-DNA-S-adenosyl-homocysteine complex, computational model [23]. The models were rendered by PyMOL Molecular Graphic System.

Biochemical and genetic studies revealed many details of the mechanisms of DNA recognition and methyl transfer by M.SssI [25–29]. From a structural point of view M.MpeI is better characterized. There is an X-ray structure of an M.MpeI-DNA complex [30], whereas for M.SssI only a computational model of an M.SssI-DNA complex is available [23]. The crystal structure of the M.MpeI complex as well as the computational model of the M.SssI complex show that the two enzymes have the common fold characterizing C5-MTases (Fig 2). The enzymes are composed of two domains separated by a DNA binding cleft and appear to interact with the DNA in a similar manner. Motifs I-VIII and part of motif X fold into the large domain, whereas the small domain contains motifs IX and the TRD [23, 30].

Both M.SssI [19, 31, 32] and M.MpeI (our unpublished observations) show the interesting phenomenon of fragment complementation, *i*.*e*. some truncated inactive fragments of the enzymes can assemble to form active MTase when expressed in the same *E*. *coli* cell. The capacity of M.SssI and M.MpeI for fragment complementation offers promising approaches for targeted DNA methylation [19]. When we tried to separately express and purify the complementing fragments for *in vitro* studies, most fragments, especially the C-terminal ones were hard to overexpress and/or showed low solubility. Fusing the C-terminal fragments to solubility-enhancing tags (thioredoxin, maltose binding protein) did not improve solubility or impaired complementation capacity (our unpublished observation). We assumed that poor solubility of the C-terminal fragments was due to the displacement of the hydrophobic C-terminal α-helix (Figs 2 and S1). In the native enzyme this α-helix folds back into the large domain, whereas in the separately expressed C-terminal fragments it can be, in lack of its natural environment, exposed to the solvent. We thought that this problem could be circumvented by using fragments obtained from circularly permuted variants of the enzymes, in which the C-terminal α-helix is covalently linked to the N-terminus of the enzyme. For proteins circular permutation is a rearrangement of the amino acid sequence, in which the original N- and C-termini are covalently linked and new ends are created by splitting the polypeptide chain somewhere else [33] [34]. Construction of circularly permuted and enzymatically active M.MpeI and M.SssI variants appeared feasible because in the structural models the N- and C-termini were closely located [23, 30].

Here we describe construction and initial characterization of circularly permuted variants of M.MpeI and M.SssI. We show that the majority of the CP variants have detectable MTase activity. Moreover, we show that the most active CP variants are capable of fragment complementation and that some complementing fragments are more soluble than fragments derived from the wild type enzyme.

By searching the REBASE database we identified new C5-MTases, which have circularly permuted amino acid sequence.

## Materials and methods

### Strains and growth conditions

*E*. *coli* DH10B (F − *endA1 recA1 galE15 galK16 nupG rpsL ΔlacX74 80dlacZΔM15 araD139 Δ(ara leu)7697 mcrA Δ(mrr-hsdRMS-mcrBC) relA1 spoT1* λ−) [35] was used as cloning host. Bacteria were grown in LB medium [36] at 30 or 37°C. The antibiotics ampicillin (Ap) and kanamycin (Kn) were used at 100 and 50 μg/ml, respectively. L-arabinose (Sigma) was used at 0.1%.

### Recombinant DNA techniques

Purification of plasmid DNA, restriction endonuclease digestion, agarose gel electrophoresis of DNA fragments, polymerase chain reaction and cloning of DNA fragments were carried out by standard procedures [36]. Nucleotide sequence of relevant parts of the plasmids was determined by automated DNA sequencing. Oligonucleotides were synthesized in this institute and are listed in S1 Table.

### Plasmids

The plasmids pBNH-M.SssI (Ap^R^) [31] and pET-28a::MMpe (Kan^R^) [30] were the sources of the genes encoding M.SssI and M.MpeI, respectively.

Plasmid pBAD24 (Ap^R^) [37] and its Kn^R^, ColE1-compatible derivative pOK-BAD [38] were used as expression vectors. Transcription of the target genes cloned in these plasmids is controlled by the *E*. *coli araBAD* promoter and the AraC protein, and can be induced by arabinose and repressed by glucose [37, 38].

To construct a tandemly duplicated M.MpeI gene, the M.MpeI coding sequence was PCR-amplified using pET-28a::MMpe as template and AK387 and AK388 as primers (S1 Table). The primers added XhoI sites to the ends of the amplified DNA fragment. The PCR product was digested with XhoI and cloned into the unique XhoI site located at the 3’-end of the M.MpeI gene in pET-28a::MMpe. The resulting plasmid (pET-tdM.MpeI) contains two tandemly arranged copies of the M.MpeI gene fused in frame.

A plasmid containing the duplicated M.SssI gene was constructed by PCR synthesis of the M.SssI coding sequence using pBNH-M.SssI as template and AK413 and AK414 as primers (S1 Table). The primers introduced an upstream NcoI site and a downstream XhoI site into the PCR product. The synthesized fragment was digested with NcoI and XhoI, and cloned between the unique NcoI and XhoI sites of pBNH-MSssI to create pB-tdM.SssI. In plasmid pB-tdM.SssI the two copies of the M.SssI gene are fused in frame.

Plasmids expressing circularly permuted variants of M.MpeI and M.SssI were constructed by PCR using pET-tdM.MpeI or pB-tdM.SssI as template, and primers listed in Tables 1, 2 and S1. The forward primers contained an in-frame ATG codon, whereas the reverse primers contained the complement of an in-frame stop codon (S1 Table). In some cases a GGT triplet (Gly), was added after the start codon. To facilitate cloning of the PCR products, the PCR primers contained restriction sites as 5’-extensions (S1 Table). The PCR products were cloned in pBAD24 [37].

**Table 1:** Plasmids expressing circularly permuted variants of M.MpeI.

Plasmid	Primers used for PCR^1^	N-terminal extension^2^	P. E. values^3^	MTase activity *in vivo*^4^
pcp35M.MpeI	AK415, AK416	MG	0.772	++
pcp62M.MpeI	AK417, AK418	MG	0.823	+++
pcp122M.MpeI	AK419, AK420	MG	0.781	+
pcp192M.MpeI	AK442, AK443	MG	0.752	+
pcp208M.MpeI	AK444, AK445	MG	0.602	+/-
pcp215M.MpeI	AK461, AK462	MG	0.328	+/-
pcp222M.MpeI	AK452, AK453	MG	0.647	-
pcp245M.MpeI	AK393, AK394	M	0.768	++
pcp280M.MpeI	AK395, AK396	M	0.676	++
pcp332M.MpeI	AK421, AK422	M	0.680	++
pcp351M.MpeI	AK423, AK424	MG	0.570	++
pcp357M.MpeI	AK397, AK398	M	0.305	-
pcp361M.MpeI	AK425, AK426	MG	0.745	++
pcp377M.MpeI	AK391, AK392	M	0.214	-
pcp62M.MpeI-280	AK417, AK418	MG	0.823	+++

^1^Nucleotide sequences of the primers are shown in S1 Table.

^2^Amino acid(s) added to the N-terminus of the variant as a result of the cloning procedure.

^3^Calculated using the CPred program.

^4^Estimated from the resistance of the plasmids to digestion with methylation-sensitive restriction enzymes (see below).

**Table 2:** Plasmids expressing circularly permuted variants of M.SssI.

cpM.SssI variant	Primers used for PCR^1^	N-terminal extension^2^	P. E. values^3^	MTase activity *in vivo*^4^
pcp33M.SssI	AK431, AK432	M	0.772	++
pcp58M.SssI	AK448, AK449	MG	0.823	+++
pcp156M.SssI	AK433, AK434	MG	0.685	++
pcp173M.SssI	AK435, AK436	MG	0.531	++
pcp243M.SssI	AK428, AK429	M	0.768	++
pcp308M.SssI	AK437, AK438	M	0.419	-
pcp357M.SssI	AK446, AK447	M	0.745	++
pcp58M.SssI-280	AK448, AK449	MG	0.823	+++

^1^Nucleotide sequences of the primers are shown in S1 Table.

^2^Amino acid(s) added to the N-terminus of the variant as a result of the cloning procedure.

^3^Calculated by the CPred program for the corresponding amino acid of M.MpeI.

^4^Estimated from the resistance of the plasmids to digestion with methylation-sensitive restriction enzymes (see below).

To obtain the cp62M.MpeI-280 variant, which differs from cp62M.MpeI by a linker peptide (GGGSG) separating the native N- and C-termini, the AK280-AK281 duplex (S1 Table) was cloned into the XhoI site located between the two M.MpeI gene copies in pET-tdM.MpeI. The resulting plasmid (pET-tdM.MpeI-280) served as template for PCR synthesis of the cp62M.MpeI-280 gene, which was cloned in pBAD24 to obtain pcp62M.MpeI-280 (Table 1). The plasmid expressing the equivalent M.SssI variant (pcp58M.SssI-280) was constructed by directly cloning the AK280-AK281 duplex into the unique XhoI site of pcp58M.SssI (Table 2).

Plasmids expressing fragments of M.MpeI or M.SssI (Table 3) were constructed by PCR synthesis of the corresponding gene segments and cloning the PCR products either in pBAD24 or pOK-BAD. The templates and primers used for the PCR synthesis are listed in S2 Table.

**Table 3:** Complementation between truncated inactive fragments of M.MpeI and M.SssI *in vivo*.

plasmid (vector: pBAD24)	plasmid (vector: pOK-BAD)	complementation *in vivo*^*1*^
pB-Mpe[361–244]	pOB-Mpe[245–360]	-
pB-Mpe[1–61]	pOB-Mpe[62–395]	+++
pB-Mpe[192–61]	pOB-Mpe[62–191]	-
pB-Mpe[280–61]	pOB-Mpe[62–279]	++
pB-Mpe[192–61]	pOB-Mpe[62–279]	++
pB-Sss[1–57]	pOB-Sss[58–386]	-
pB-Sss[357–242]	pOB-Sss[243–356]	-
pB-Sss[58–242]	pOB-Sss[243–57]	-
pB-Sss[58–275]	pOB-Sss[276–57]	+/-
pB-Sss[58–275]	pOB-Sss[243–57]	+

^1^DNA methyltransferase activity was estimated by purifying plasmid DNA from co-transformed cells and digesting it with the methylation sensitive restriction enzyme Hin6I (S5 and S6 Figs).

### Enzymes and chemicals

Restriction endonucleases, T4 DNA ligase and Phusion DNA polymerase were purchased from Thermo Scientific or New England Biolabs. S-adenosyl-L-[methyl-^3^H]methionine ([methyl-^3^H]-SAM) was purchased from PerkinElmer and unlabeled S-adenosyl-L-methionine from New England Biolabs.

### Estimation of DNA methyltransferase activity

Methyltransferase activity in *E*. *coli* was routinely estimated by restriction protection assay. Cells with the plasmid encoding the MTase variant to be tested were grown in LB/Ap medium to OD_600_~0.5, then production of the MTase was induced by adding 0.1% arabinose, and growth was continued for 5 hours at 30°C. Plasmid DNA extracted from the cultures was digested with the CG-specific methylation-sensitive restriction endonucleases Hin6I and/or Eco47I. Hin6I recognizes the 5’-GCGC-3’ sequence but does not cut when the underlined cytosines are methylated: 5’-GCGC/5’-GCGC (Kazlauskiene et al., cited in REBASE[15]). Eco47I recognizes the sequence 5’-GGWCC. The hemimethylated recognition site (5’-GGWCC/5’-GGWCC) is resistant to cleavage (Kazlauskiene et al., cited in REBASE [15]).

To test complementation between split MTase fragments *in vivo*, segments of the M.MpeI and M.SssI genes were cloned in pBAD24 or pOK-BAD as described above. Pairs of plasmids carrying different parts of the same MTase gene in pBAD24 and pOK-BAD vector were co-transformed into *E*. *coli* DH10B. Induction of MTase fragment production by arabinose and analysis of the methylation status of the plasmid preparations were done as described above for the full-length enzymes.

Methyltransferase activity was measured *in vitro* in crude extracts using S-adenosyl-L-[methyl-^3^H]methionine. Cultures were grown to a density of OD_600_ ~ 0.5, then MTase production was induced by adding 0.1% arabinose, and growth was continued for 5 hours at 30°C. Cells from 18 ml were harvested by centrifugation, resuspended in 2 ml of a buffer containing 50 mM Tris-HCl pH8.0, 300 mM NaCl, 5% glycerol, and disrupted by sonication. Cell debris was removed by centrifugation in a bench-top centrifuge (13,000 rpm, 3 min at 4°C). MTase activity was measured using λ phage DNA (Thermo Fisher) and [methyl-^3^H]SAM essentially as described previously [31].

### SDS-polyacrylamide gel electrophoresis

Cell extracts were prepared as described above and proteins were analyzed by SDS-polyacrylamide gel electrophoresis using conventional SDS-polyacrylamide gels [36]. Solubility of the protein of interest was estimated by comparing coomassie blue stained samples from total cell extracts and from supernatants obtained after removal of the cell debris by centrifugation (13,000 rpm, 3 min at 4°C).

### Bioinformatics tools

Permutation sites were designed by CPred [39]. Amino acid sequences were aligned using the BLAST [40] or the CLUSTAL Omega at EBI [41] programs. Protein structures were visualized by PyMol (The PyMOL Molecular Graphics System, Version 1.8 Schrödinger, LLC). Hydropathy values were generated by the EMBOSS Pepinfo tool (https://www.ebi.ac.uk/Tools/seqstats/emboss_pepinfo) using using Kyte and Doolittle parameters [42].

Circularly permuted C5-MTases in the REBASE database [15] were identified by a computer-aided search of 20,677 entries using the criterion of motif X to precede motif I in the amino acid sequence. The search was implemented using the Biopython tools [43] (see S1 Appendix). Each sequence found in the search was checked manually to exclude false positives.

## Results

### Design and construction of circularly permuted MTase variants

**Fig 3:**
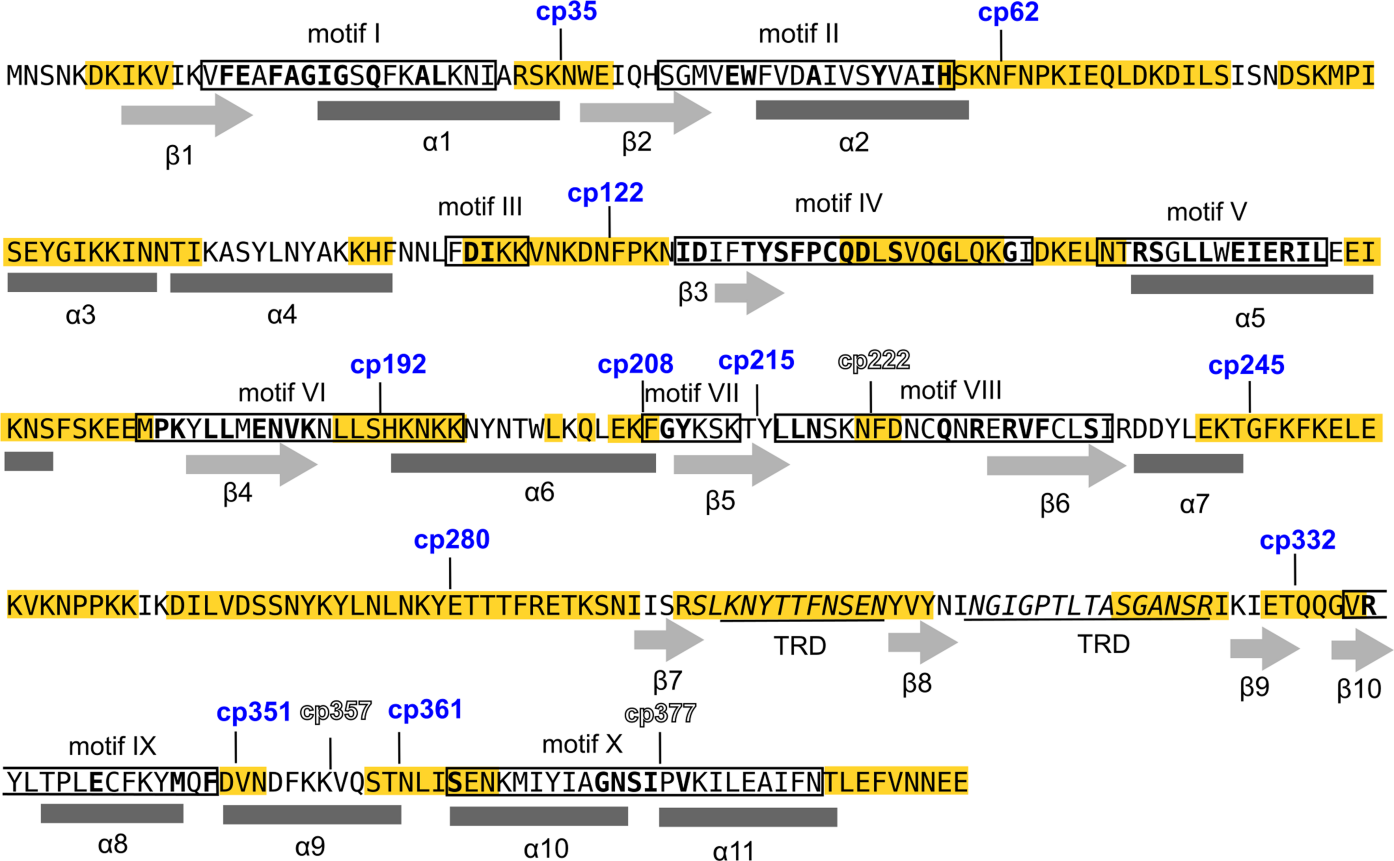
Split sites of circularly permuted M.MpeI variants. The new N-termini of the active and inactive variants are indicated above the sequence by blue and outlined numbers, respectively. Conserved motifs are boxed, α-helices are marked under the sequence by rectangles and β-strands by arrows [30]. The most conserved residues are printed in bold. Regions predicted by the CPred program [39] to contain viable permutation sites are highlighted in yellow.

**Fig 4:**
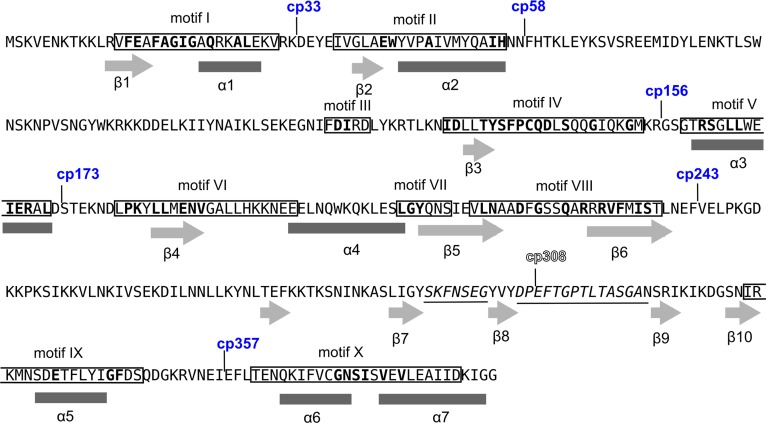
Split sites of circularly permuted M.SssI variants. The new N-termini of the active and inactive variants are indicated above the sequence by blue and outlined numbers, respectively. The predicted secondary structural elements [23] are shown under the sequence: rectangles, α-helices; arrows, β-strands. The most conserved residues are printed in bold.

In the crystal structure of the M.MpeI-DNA-S-adenosyl-homocysteine complex (PDB: 4DKJ) as well as in the computational model of the M.SssI ternary complex the N- and the C-termini of the enzymes are located closely in space, which encouraged us to construct circularly permuted variants. Permutation sites were designed with the web-based tool Circular Permutation Site Predictor (CPred) [39]. CPred uses the 3D structure of the protein-of-interest as an input and assigns a Probability Estimate (P. E.) to each amino acid of the molecule. Residues with P. E. values above 0.5 are considered by the program viable permutation sites. The distribution of viable and non-viable cleavage sites along the peptide chain of M.MpeI is shown in Fig 3. Most of the predicted viable permutation sites are clustered in blocks which, as one would expect, coincide with non-conserved regions. Because of insufficient quality in some regions, the computational model of M.SssI was not accepted by CPred. To select permutation sites for M.SssI (Fig 4), we used the P. E. values calculated for M.MpeI, and identified the corresponding residues in M.SssI by sequence alignment. To minimize the possibility of disrupting the native structure, most split sites were designed to fall outside of conserved motifs and the target recognition domain (Figs 3 and 4).

Plasmids encoding circularly permuted variants of M.MpeI and M.SssI were constructed using the concatamerization strategy [33, 44] (S2 Fig). First in-frame fusions were created between tandemly duplicated copies of the MTase genes and cloned to obtain plasmids pET-tdM.MpeI and pB-tdM.SssI. In pET-tdM.MpeI the 3’-end of the first copy is directly connected to the 5’-end of the second copy of the M.MpeI gene (S1 Dataset), whereas in pB-tdM.SssI a short sequence encoding the LEC tripeptide separates the two M.SssI gene copies (S2 Dataset). The duplicated MTase genes served as templates for the PCR-synthesis of the circularly permuted MTase genes (S2 Fig). The PCR fragments were cloned in the expression vector pBAD24 [37] downstream of the arabinose-inducible P_BAD_ promoter of *E*. *coli* (S2 Fig). The plasmids and the encoded cpMTase variants were named to indicate the new N-terminal amino acid (*e*.*g*. cp192M.MpeI starts with Gly192 and ends with Ser191 of wild-type M.MpeI). The amino acid sequences of the permuted MTases are shown in S1 and S2 Datasets.

To explore the permutation potential of the MTases, variants with split sites distributed over the whole molecule were created. Plasmids encoding fourteen M.MpeI and seven M.SssI permutants were constructed (Tables 1 and 2). Collectively, the permutation sites in the two enzymes represent each segment separating the adjacent conserved motifs (Figs 3 and 4).

### Methyltransferase activity of the circularly permuted MTases

MTase activity was first tested by a restriction protection assay as described in Materials and Methods. Plasmids encoding wild-type M.MpeI or M.SssI were almost completely protected against Hin6I digestion even if the plasmid was isolated from uninduced cells. Plasmids expressing the tandemly duplicated M.MpeI or M.SssI (pET-tdM.MpeI and pB-tdM.SssI) showed similar level of resistance as the plasmids expressing wild-type M.MpeI or M.SssI (not shown), suggesting that the activity of the fused dimers was comparable to that of the wild-type enzymes. Previously, similar observations were made with the tandemly duplicated variant of another C5-MTase, M.HaeIII [14].

**Fig 5:**
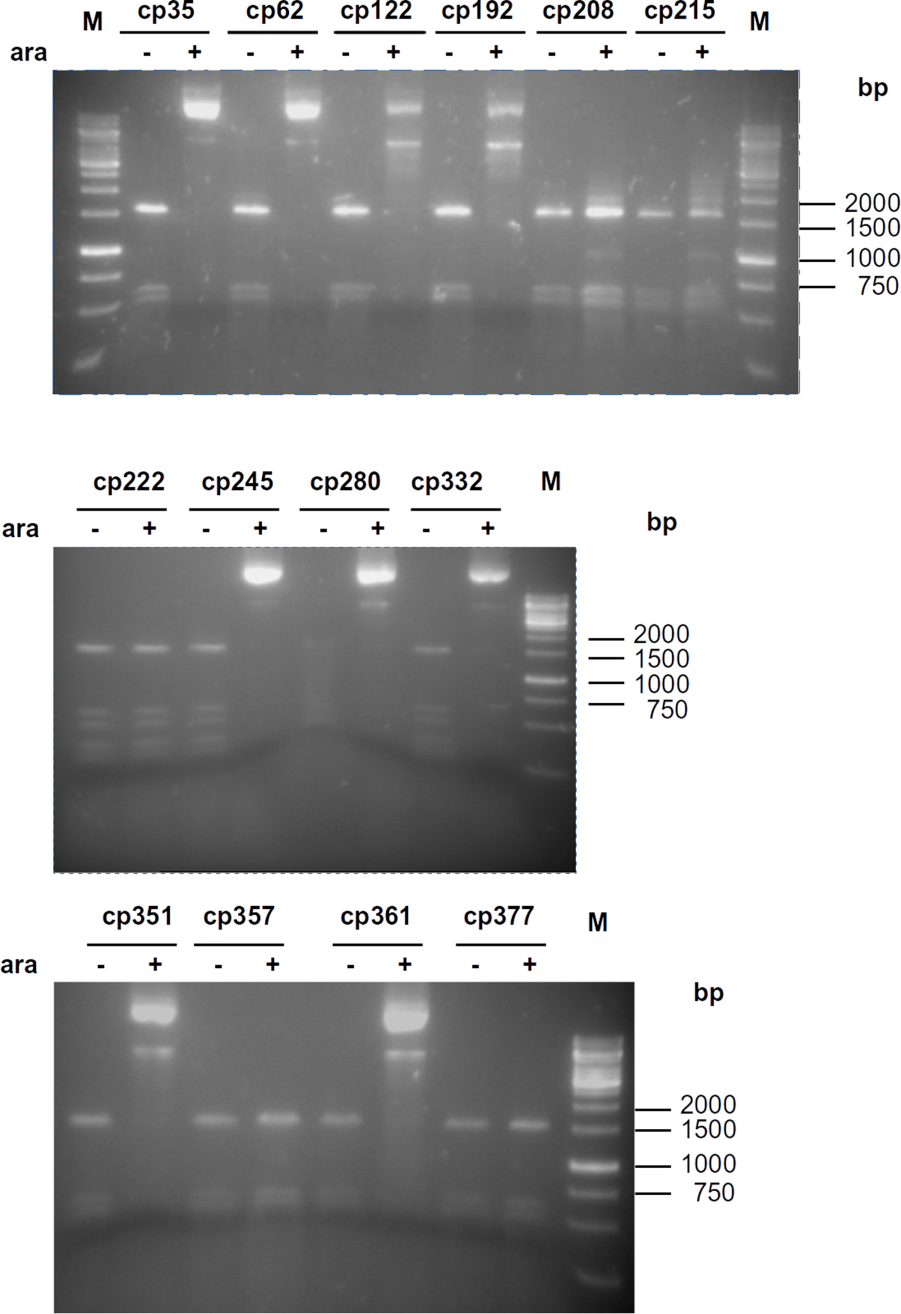
Estimation of DNA methyltransferase activity of circularly permuted M.MpeI variants by restriction protection assay. Plasmids were digested with Hin6I restriction endonuclease. Plasmids purified from arabinose-induced or uninduced cultures are indicated by + or - signs, respectively. M, GeneRuler 1 kb DNA Ladder.

Of the plasmids encoding cpM.MpeI variants, pcp222M.MpeI, pcp357M.MpeI and pcp377M.MpeI were fully digestible even after arabinose-induction indicating that cp222M.MpeI, cp357M.MpeI and cp377M.MpeI had no detectable MTase activity (Fig 5). The other plasmids showed different degrees of protection ranging from the appearance of faint bands (pcp208M.MpeI, pcp215M.MpeI) to full or almost full protection after induction ((cp35M.MpeI, cp62M.MpeI, cp245M.MpeI, cp280M.MpeI or cp332M.MpeI). Plasmid pcp62M.MpeI showed slight protection even when it was purified from uninduced cells, suggesting that of all circularly permuted M.MpeI variants cp62M.MpeI had the highest MTase activity (Fig 5). Because the *araBAD* promoter is tightly controlled [37], the MTase activity of cp62M.MpeI detected in the uninduced state probably indicates read-through transcription from an upstream promoter in the plasmid.

In some experiments the restriction enzyme Eco47I was used to detect CG-specific methylation. Eco47I recognizes GGWCC sites but can not cleave when the underlined cytosine is methylated (Kazlauskiene et al., cited in REBASE[15]). At one of the Eco47I sites in the pcpM.MpeI and pcpM.SssI plasmids the 3’-C is followed by a G creating an M.SssI/M.MpeI substrate site. Methylation of this CG site blocks Eco47I cleavage and yields a 1059 bp protected fragment. The results of Eco47I digestion were in agreement with the results obtained with Hin6I digestion: the 1059 bp fragment was detectable in the digests of all plasmids showing some level of protection against Hin6I digestion, but was missing from the digests of pcp222M.MpeI, pcp357M.MpeI and pcp377M.MpeI, which were fully digestible with Hin6I. Moreover, similarly to the Hin6I digestion, the plasmid pcp62M.MpeI showed slight resistance to Eco47I digestion even in the uninduced state (S3 Fig).

**Fig 6:**
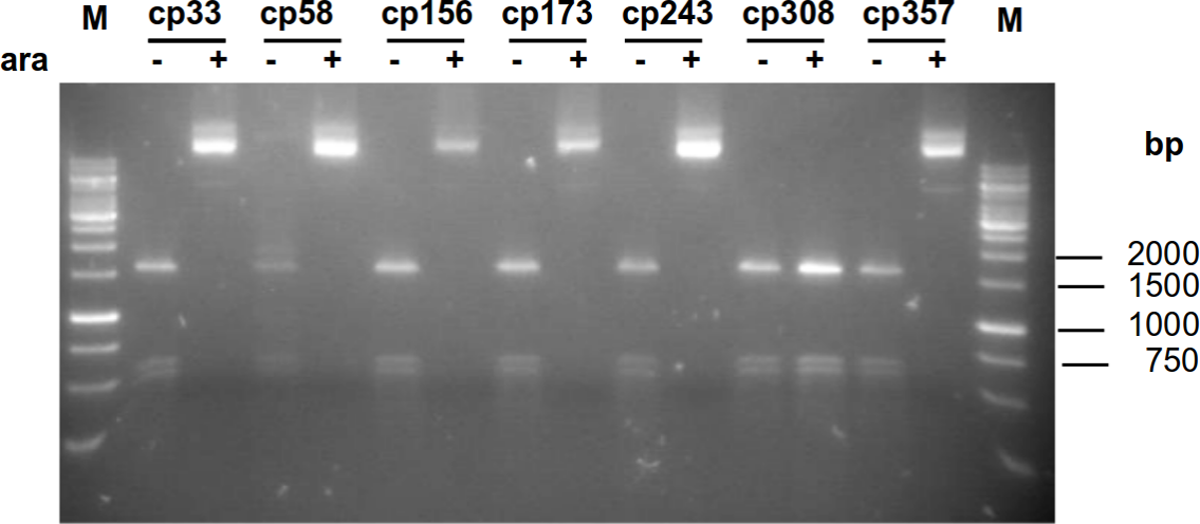
Estimation of DNA methyltransferase activity of circularly permuted M.SssI variants by restriction protection assay. Plasmids were digested with Hin6I restriction endonuclease. Plasmids purified from arabinose-induced or uninduced cultures are indicated by + or - signs, respectively. M, GeneRuler 1 kb DNA Ladder.

Of the seven circularly permuted M.SssI variants cp308M.SssI was inactive (Fig 6). Plasmids encoding cp33M.SssI, cp58M.SssI, cp156M.SssI, cp173M.SssI, cp243M.SssI and cp357M.SssI were resistant to Hin6I digestion when they were purified from arabinose-induced cells indicating that these CP variants had MTase activity. The plasmid expressing cp58M.SssI showed some protection even in the uninduced state (Figs 6 and S4). The high activity of cp58M.SssI *in vivo* was not surprising because its permutation site corresponded to that of cp62M.MpeI, which was the most active circular permutant of M.MpeI (Fig 1, Tables 1 and 2).

MTase activity of the CP variants was also estimated in cell extracts by a radioactive assay. The activity measured in the cp62M.MpeI extract was comparable to that of the WT enzyme, whereas the activities measured in the extracts of other cpM.MpeI and cpM.SssI variants were much lower (S12 and S13 Figs). The low activity of cp58M.SssI was especially unexpected because the methylation state of the plasmid encoding cp58M.SssI indicated high activity (see above).

The crystal structure of M.MpeI [30] as well as the computational model of M.SssI [23] suggested that in both MTases the N- and C-termini are close to each other. However, because a few terminal amino acids are missing from the M.MpeI X-ray model (the terminal residues in the model are D6 and N293), we could not determine the exact distance between the two ends of the molecule. Thus it was not possible to exclude that linking the ends would lead to structural perturbations affecting MTase activity. We inserted a flexible linker peptide (GGGSG) between the native N- and C-termini of cp62M.MpeI and cp58M.SssI (S1 and S2 Datasets). The plasmids pcp62M.MpeI-280 and pcp58M.SssI-280 expressing the linker-containing MTases showed the same level of resistance to Eco47I digestion as the respective parental plasmids (pcp62M.MpeI and pcp58M.SssI, suggesting that direct fusing of the native N- and C-termini had no adverse effect on the catalytic activity. The cpM.MpeI and cpM.SssI variants are listed in Tables 1 and 2, respectively.

**Fig 7:**
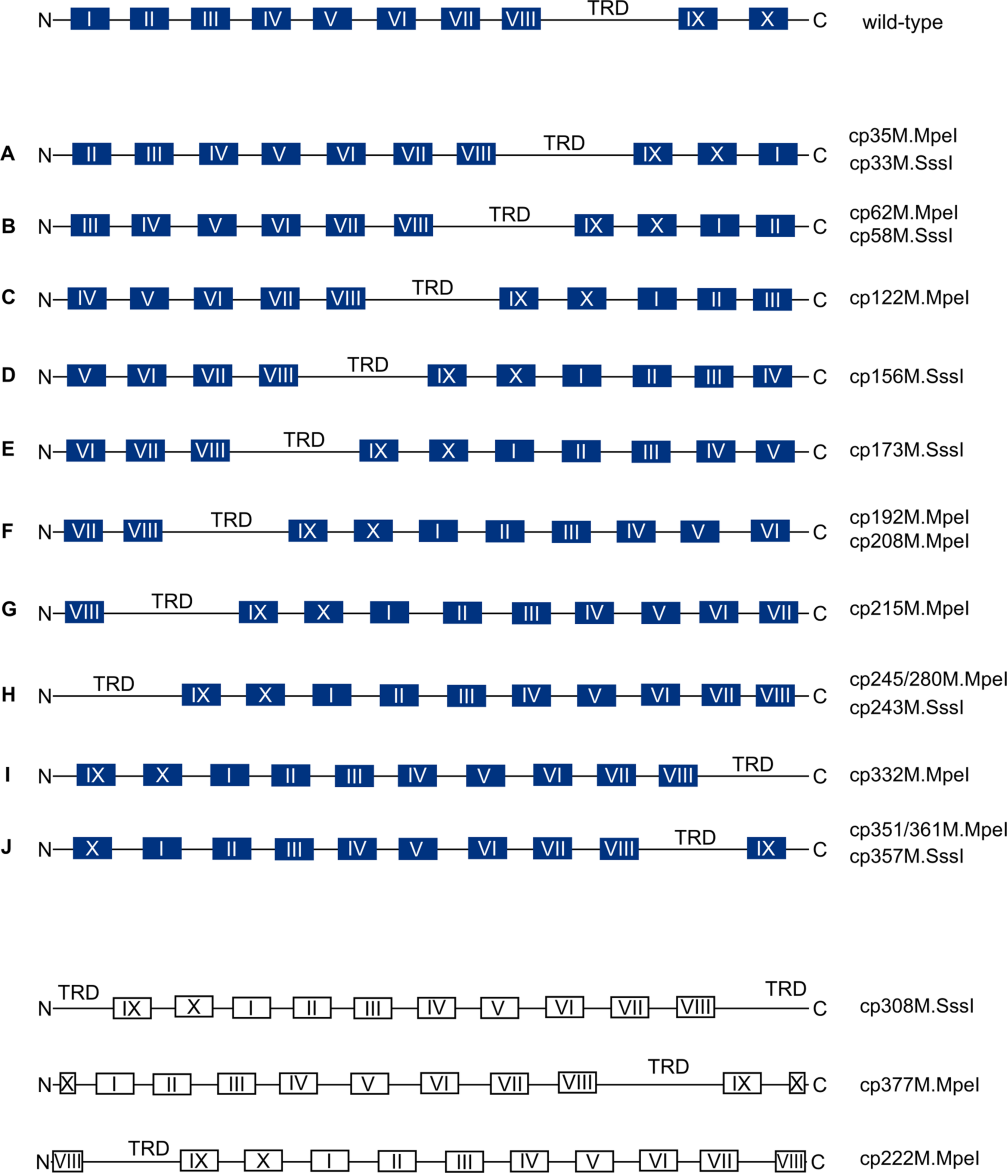
Schematic representation of the circularly permuted MTase variants constructed in this work. Conserved motifs characteristic for C5-MTases [3, 6] are marked with roman numerals. TRD, target recognizing domain. Variants with detectable MTase activity are shown with filled boxes and variants lacking MTase activity (grouped at the bottom of the figure) with empty boxes.

Based on the position of the permutation sites, the permutants possessing detectable MTase activity can be classified in ten types (A through J, Fig 7).

### Complementation between fragments of circularly permuted MTases

This work was started on the hypothesis that poor solubility of C-terminal fragments of M.MpeI and M.SssI was due to the exposure of the C-terminal α-helix to the solvent, and that the native fold of the split fragments could be better preserved by covalently linking the C-terminal α-helix to the large domain. Of all permutants we have constructed, Class J variants appeared to be the best starting material for creating complementing fragments, because splitting them between motif VIII and the TRD would create two polypeptides, which approximately correspond to the two domains of the native enzyme (Fig 7). Consistently with the interdomain position of the split site, C5-MTases bisected naturally [45, 46] or artificially [24, 31, 47] between motif VIII and the TRD, showed efficient fragment complementation.

**Fig 8:**
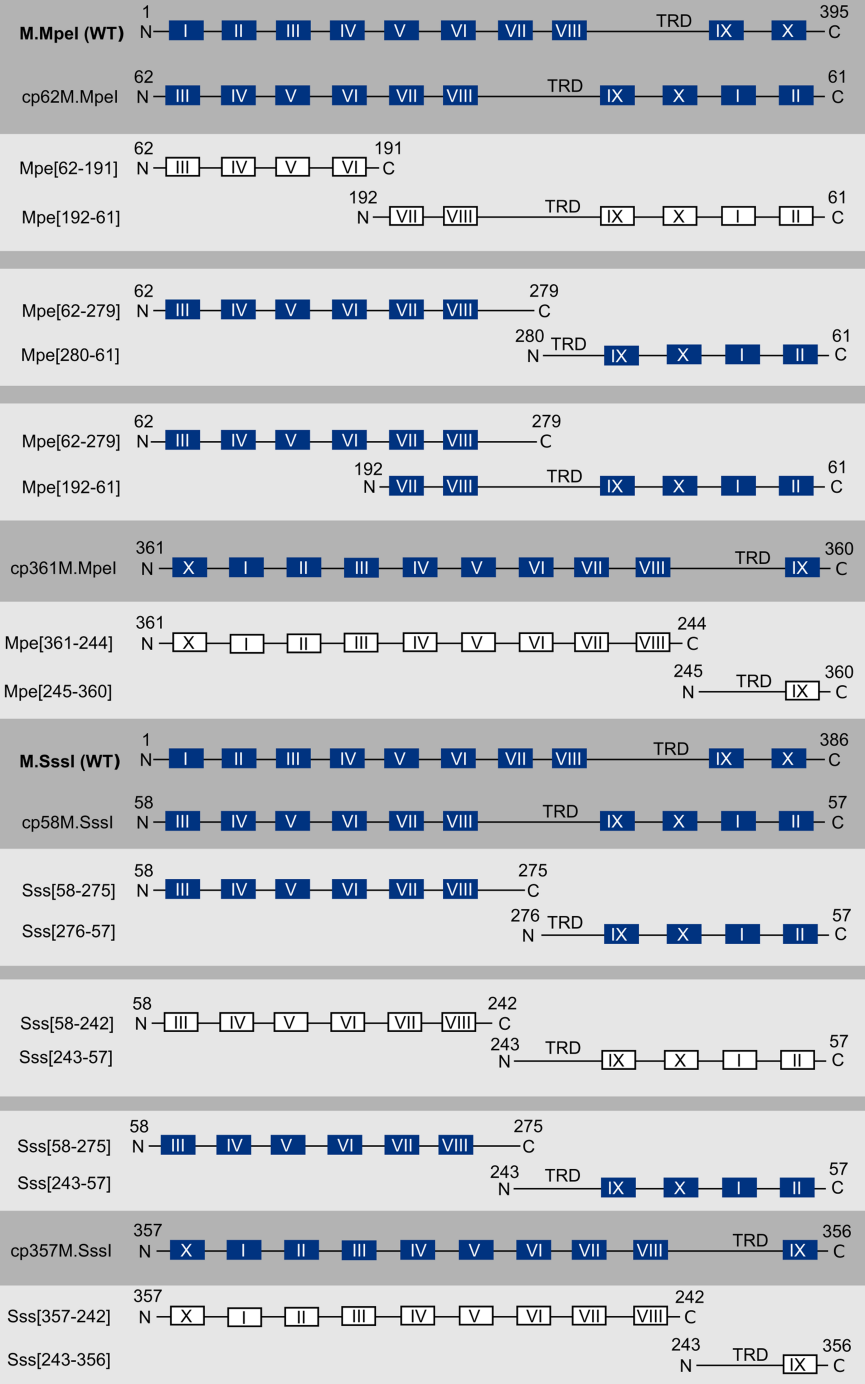
Scheme of the fragments obtained from circularly permuted variants of M.MpeI and M.SssI. Full-length variants and pairs of complementing fragments having MTase activity are shown with filled boxes, whereas fragment pairs that do not have MTase activity are depicted with empty boxes.

The N- and C-terminal halves of the cp361M.MpeI and cp357M.SssI genes were cloned separately in the compatible plasmid vectors pBAD24 and pOK-BAD to yield pB-Mpe[361–244], pOB-Mpe[245–360], pB-Sss[357–242] and pOB-Sss[243–356]. The plasmids express, in arabinose-inducible fashion, the fragments specified by the numbers in square brackets (Tables 3 and S2, Figs 8, S3 and S4 Datasets).

Complementation between plasmids carrying different segments of a MTase genes was tested by co-transforming *E*. *coli* DH10B with the plasmid pair, and analyzing the methylation status of the plasmid DNA purified from double-transformants. Plasmids prepared from cells containing pB-Mpe[361–244] + pOB-Mpe[245–360] as well as the plasmids purified from cells containing pB-Sss[357–242] + pOB-Sss[243–356] were completely digestible with Hin6I even if they were extracted from arabinose-induced cultures. These results indicated that the tested fragment pairs did not form functional MTase.

After the failure with Class J, we tried Class B permutants for complementing fragments. In Class B enzymes (cp62M.MpeI and cp58M.SssI, Fig 7) the new N-terminus is between conserved motifs II and III. Of all permutants Class B variants showed the highest MTase activity. First we tested whether fragments created by spliting WT M.MpeI or M.SssI between motifs II and III have complementation capacity. We constructed the compatible plasmids pB-Mpe[1–61] and pOB-Mpe[62–395] expressing the indicated fragments from the *E*. *coli araBAD* promoter (Tables 3 and S2, Fig 8 and S3 Dataset). The plasmid DNA prepared from induced cells containing both plasmids was almost completely resistant to Hin6I indicating that the M.MpeI fragments [1–61] and [62–395] could form active MTase (S5 Fig, Table 3). Surprisingly, the equivalent fragments of M.SssI ([1 – 57] + [58–386]) did not show MTase activity (S6 Fig, Table 3).

To test the complementation potential of the fragments derived from cp62M.MpeI, two plasmid pairs were constructed. The plasmids pB-Mpe[192–61] and pOB-Mpe[62–191] express fragments split at H192 in conserved motif VI, whereas the plasmids pB-Mpe[280–61] and pOB-Mpe[62–279] express fragments bisected at E280, in the region between motif VIII and the TRD (Table 3, Figs 1 and 8). The plasmid pair pB-Mpe[192–61] and pOB-Mpe[62–191] did not complement. In contrast, pB-Mpe[280–61] and pOB-Mpe[62–279] purified from arabinose-induced cells were partially protected against Hin6I digestion indicating that the Mpe[280–61] and Mpe[62–279] peptides derived from cp62M.MpeI formed active MTase (S5 Fig, Table 3).

The complementation capacity of cp58M.SssI, the Class B permutant version of M.SssI was tested in similar experiments. The permutation site of cp58M.SssI exactly corresponds to that of cp62M.MpeI (Fig 1). Two bisection sites were tested, both were designed to fall between conserved motif VIII and the TRD. The split site at V243 determined the fragments Sss[58–242] and Sss[243–57], whereas the one at N276 determined Sss[58–275] and Sss[276–57] (Figs 1 and 8). When tested for complementation in *E*. *coli*, the fragment pair Sss[58–242] + Sss[243–57] was found to be inactive, whereas the Sss[58–275] + Sss[276–57] fragments showed very weak MTase activity (S6 Fig, Table 3).

The experiments described above analyzed complementation ability of fragments, which were precise cleavage products of the parental cpMTase without gaps or extraneous amino acids. Because fragments of wild-type M.SssI and M.MpeI showed efficient complementation even if the fragments had long overlapping segments ([31] and our unpublished observation), it was interesting to test whether the circularly permuted variants have the same capacity. Plasmids pB-Mpe[192–61] and pOB-Mpe[62–279], which produce fragments with an 87 amino acid overlap (Fig 8 and S3 Dataset) were partially protected against Hin6I digestion (S5 Fig, Table 3). The fragment pair Sss[58–275] + Sss[243–57] having a 32 amino acid overlap (Fig 8 and S4 Dataset) had very weak complementation ability (S6 Fig, Table 3).

### Solubility of circularly permuted MTases and their fragments

Production and solubility of CP variants, which had detectable MTase activity *in vivo* was investigated by SDS-polyacrylamide gel electrophoresis of extracts prepared from uninduced and arabinose-induced *E*. *coli* cells. For most cpM.MpeI variants the amounts of the MTase detected by SDS gel electrophoresis correlated with the *in vivo* activities: WT M.MpeI, cp62M.MpeI, cp245M.MpeI and cp351M.MpeI, which were highly active *in vivo* (Fig 5), were produced upon induction in relatively large amounts and were soluble, whereas cp122M.MpeI, cp208M.MpeI and cp215M.MpeI, which had low activity, were not detectable on the gels (S7 Fig). Three variants (cp35M.MpeI, cp280M.MpeI and cp332M.MpeI) were active *in vivo* and were produced in detectable amounts but showed poor solubility (S7 Fig). The M.SssI variants including the WT enzyme were produced in lower amounts than the M.MpeI variants (S8 Fig).

Production and solubility of complementing M.MpeI fragments was tested by separately expressing the fragments in *E*. *coli*. The [192–61] plus [62–279] combination were the only fragment pair, which showed complementation, were produced in relatively large amounts, and were at least partially soluble (S9 Fig).

### Natural circularly permuted C5-MTases

Based on the linear order of conserved motifs, the CP variants showing MTase activity represented ten topological types (Fig 7). Type H, in which the new amino-end is between motif VIII and the TRD, has already been observed in five natural C5-MTases: M.BssHII [11],[12], M.Alw26I, M2.Eco31IC, M.Esp3I [13] and M2.BsaI [14] and observations by Zhu and Xu, cited in REBASE [15]). To explore if there are natural circularly permuted C5-MTases with permutation topology different from Type H, we performed a computer search of the C5-MTase sequences available in the REBASE database. The computer search found 27 C5-MTase sequences satisfying the criterion of motif X preceding motif I. The sequences included the five MTases already known to have circularly permuted sequence (M.BssHII, M.Alw26I, M2.Eco31IC, M.Esp3I and M2.BsaI), and 22 new enzymes. The latter group contains four enzymes (M2.BcoDI, M.BsmAI, M2.EcoMI and M.BsmBI), for which there is biochemical evidence showing that the reaction product is C5-methylcytosine. The other 18 enzymes are putative C5-MTases (S5 Dataset) identified only by the presence of the characteristic sequence motifs. The amino acid sequence alignments show that all natural circularly permuted C5-MTases found previously or in the present search are permuted in the variable region (S5 Dataset). Because the assigment of the TRD is somewhat arbitrary, it is not easy to decide whether the natural cpMTases fall in the H or in the J class of the proposed classification. Using the assignment of the TRD for M.Alw26I, M2.Eco31IC, M.Esp3I [13], the majority of natural circularly permuted C5-MTases appear to have the Type H arrangement (S5 Dataset).

## Discussion

N6-adenine and N4-cytosine DNA MTases are rather heterogenous and can be classified on basis of the order of their functional subdomains [48–51]. In contrast, C5-cytosine MTases have a more uniform structure. In the vast majority of C5-MTases the conserved motifs are arranged in the same linear order (I through X) [3, 6]. There were a few known exceptions, enzymes in which the order of conserved motifs was circularly permuted relative to the canonical order: M.BssHII [11], [12], M.Alw26I, M2.Eco31IC, M.Esp3I [13] and M2.BsaI [14] and Zhu and Xu, cited in REBASE [15]). A recent study used directed evolution to create circularly permuted variants of a C5-MTase (M.HaeIII) [14].

Prompted by the wish to improve some physicochemical properties of the enzymes, we used a systematic approach to construct circularly permuted variants of the CG-specific C5-MTases M.MpeI and M.SssI. To our knowledge this is the first study describing the construction of designed circularly permuted variants of C5-MTases. Most CP variants created in this work (11 of 14 for M.MpeI and 6 of 7 for M.SssI) had detectable activity in *E*. *coli*. Although most permutation sites were designed to fall outside of conserved motifs, and the MTase activity of the permutants measured in cell extracts was in most cases substantially lower than that of the wild-type enzyme, the high proportion of CP variants with detectable activity is still remarkable, and is a new evidence for the structural plasticity of C5-MTases. The observed phenotypes *i*.*e*. the presence or absence of MTase activity of the CP variants were in most cases in agreement with the CPred predictions (Fig 3).

In three of the four inactive CP variants created in this work the permutation sites are in regions with known function: for cp222M.MpeI in motif VIII, for cp377M.MpeI in motif X, and for cp308M.SssI in the TRD (Figs 3 and 4). In the fourth inactive permutant (cp357M.MpeI) the permutation site is in the middle of α-helix 9 (Figs 3 and S10). There are two other permutants (cp351M.MpeI and cp361M.MpeI), whose permutation sites are at the ends of the same α-helix, but these variants have MTase activity (Figs 3 and 5). Apparently, splitting α-helix 9 in the middle (cp357M.MpeI) perturbs the structure more than splitting the helix at the edges (Figs 3 and S10). These results show the importance of α-helix 9 for M.MpeI function and demonstrate the usefulness of CP variants in the identification of functionally important elements of enzymes. Unfortunately, the region corresponding to α-helix 9 of M.MpeI is represented in poor quality in the computational model of M.SssI (S11 Fig) making comparisons between the two MTases difficult. The equivalent permutants (cp361M.MpeI and cp357M.SssI) had similar MTase activities *in vivo* (Figs 5 and 6).

Of all permutants constructed in this work Class B enzymes (cp62M.MpeI and cp58M.SssI) had the highest MTase activity (Figs 5 and 6, Tables 1 and 2) suggesting that the surface loop between conserved motifs II and III is rather tolerant to structural perturbations. This notion is also supported by the presence of the relatively long non-conserved region between motifs II and III in M.MpeI and M.SssI. This sequence is absent from many C5-MTases. Consistently with the assumed tolerance of the region between motifs II and III to stuctural changes, the complementation capacity of the [1–61] + [62–395] fragment pair was higher than that of any other M.MpeI fragment combinations we have tested.

Although the methylation status of the plasmids showed that the activity of cp58M.SssI was, similarly to the equivalent cp62M.MpeI, higher *in vivo* than that of the other M.SssI permutants (Fig 6), we could not detect elevated MTase activity in the crude extract of cp58M.SssI. We don’t know the reason of this discrepancy, it is possible that cp58M.SssI loses activity during preparation of the cell extract. The observed difference between the activities of cp62M.MpeI and cp58M.SssI was consistent with the difference between the amounts of soluble cp62M.MpeI and cp58M.SssI in crude extracts (S7 and S8 Figs). In our hands M.MpeI and its CP derivatives had higher activity and were easier to work with than M.SssI and its CP variants. It must be noted that in this work the activity of the MTase variants was mainly estimated from the methylation state of plasmid DNA purified from *E*. *coli* cells producing the enzyme. The *in vivo* MTase activity can be influenced by factors such as solubility, stability of the MTase, interaction with other proteins, thus the methylation status of the plasmid DNA may not truly reflect the differences between the catalytic activities of the variants. Strict comparison of the specific activities awaits enzymological studies with purified cpMTase variants.

It is interesting to compare the designed CP variants of M.MpeI and M.SssI with the CP variants of M.HaeIII created previously by a directed evolution strategy [14]. The M.HaeIII permutants were, due to the random nature of the experimental approach, not unit-length molecules, they either lacked a few amino acids or contained shorter or longer redundant peptides. Based on the position of their N-termini, the enzymatically active cpM.HaeIII variants fell in three groups. Members of the first group started either between conserved motifs II and III, or in motif III, or between motifs III and IV [14], thus they more or less corresponded to our Type B or Type C permutants. The N-termini of the second group were in the variable region between motif VIII and the TRD, hence these permutants can be classified as Type H. The permutation sites of the third group were in a more distal part of the variable region, within or very close to the TRD [14] (S14 Fig). The permutation topology of this last group does not match any of the ten types of active cpMTases constructed by us, instead it appears to correspond to cp308M.SssI, which was inactive (Figs 6 and S14). Although the two studies were done with different C5-MTases (M.HaeIII vs. M.MpeI/M.SssI), a comparison of the results (S14 Fig) shows that a much wider range of permutation topologies are compatible with C5-MTase activity (Fig 7) than detected in the previous study [14].

This work was motivated by the wish to create complementing fragments of M.SssI and/or M.MpeI, which are more soluble than those obtained from the wild-type enzymes. We did find a fragment pair derived from a circularly permuted M.MpeI variant (Mpe[192–61] and Mpe[62–279]), which had the capacity of complementation, and were more soluble than any other complementing fragment pairs we have tested (S9 Fig). Although it will require further work to determine whether the Mpe[192–61] and Mpe[62–279] fragments can be purified in good yield, these results already show that circular permutation can be a useful approach in engineering C5-MTases.

The search of the REBASE database identified 22 new C5-MTases with circularly permuted amino acid sequence. Although most of these are putative enzymes, *i*.*e*. we do not know whether they are active, the relatively large number of circularly permuted C5-MTase-like sequences (biochemically characterized and putative enzymes) suggests that circular permutation occured multiple times during evolution of this enzyme family. A mechanism involving gene duplication and subsequent bidirectional truncation was proposed to account for the evolution of circularly permuted DNA methyltransferases [52]. The permutation-by-duplication model received later experimental support [14]. The close distance of the N- and C-termini in the available structural models of C5-MTases [9, 10, 23, 30] is consistent with circular permutation as a possible mechanism in C5-MTase evolution. It will be interesting to test whether the ability to tolerate circular permutation without major loss of activity is a general phenomenon of C5-MTases.

In all natural circularly permuted C5-MTases the permutation site falls in the variable region (S5 Dataset). In this work we showed for two C5-MTases that the activity Type B permutants was comparable to that of the wild-type enzymes. Perhaps surprisingly, the Type B permutation pattern does not seem to occur in natural C5-MTases. It is possible that the Type B CP arrangement results in decreased SAM binding affinity, which could be a disadvantage *in vivo* at physiological SAM concentrations, and would explain the apparent lack of natural Type B circularly permuted C5-MTases. Under the *in vitro* assay conditions used in this work (crude extract, excess SAM) a moderate decrease in SAM binding affinity would not be detectable. To address this question we will determine the steady state kinetic constants of purified wild-type M.MpeI and cp62M.MpeI.

The uniformity of the permutation patterns in natural circularly permuted C5-MTases suggests that evolutionary pathways leaving the two domains intact are favored by Nature.

## Supporting information

S1 Appendix Search of the REBASE database for circularly permuted C5-MTases. (DOCX)

S1 Dataset Amino acid sequences of circularly permuted M.MpeI variants. (DOCX)

S2 Dataset Amino acid sequences of circularly permuted M.SssI variants. (DOCX)

S3 Dataset Amino acid sequences of cpM.MpeI fragments. (DOCX)

S4 Dataset Amino acid sequences of cpM.SssI fragments. (DOCX)

S5 Dataset Clustal O alignment of the amino acid sequences of natural circularly permuted C5-MTases. (DOCX)

S1 Fig Hydrophobicity plot of M.MpeI and M.SssI. The hydropathy values of the residues located in the C-terminal α-helix are plotted in red. The plots were generated by the EMBOSS Pepinfo service (https://www.ebi.ac.uk/Tools/seqstats/emboss_pepinfo/) using Kyte and Doolittle parameters [42].
(TIF)

S2 Fig Scheme of construction of circularly permuted MTase variants. (TIFF)

S3 Fig Eco47I digestion of plasmids encoding cpM.MpeI variants. The plasmids contain five Eco47I sites. Uninduced and arabinose-induced cultures are indicated by minus and plus signs, respectively. Appearance of a 1059 bp fragment indicates methylation of a CG site overlapping one of the Eco47I sites in the plasmid. M, GeneRuler 1 kb DNA Ladder.
(TIF)

S4 Fig Eco47I digestion of plasmids encoding cpM.SssI variants. The plasmids contain six Eco47I sites. Uninduced and arabinose-induced cultures are indicated by minus and plus signs, respectively. Appearance of a 1059 bp fragment indicates methylation of a CG site overlapping one of the Eco47I sites in the plasmid. M, GeneRuler 1 kb DNA Ladder.
(TIF)

S5 Fig Complementation between fragments of cpM.MpeI. Plasmids were digested with Hin6I. Lane 1, pB-Mpe[1–61] Lane 2, pOB-Mpe[62–395] Lane 2, pOB-Mpe[62–395] Lane 3, pOB-Mpe[62–279] Lane 3, pOB-Mpe[62–279] Lane 4, pB-Mpe[280–61] Lane 5, pB-Mpe[192–61] Lanes 6 and 7, pB-Mpe[1–61] + pOB-Mpe[62–395] Lanes 8 and 9, pB-Mpe[280–61] + pOB-Mpe[62–279] Lanes 10 and 11, pB-Mpe[192–61] pOB-Mpe[62–279] M, 1 kb GeneRuler Induced cultures were grown in the presence of 0.1% arabinose at 30°C for 5 hours.
(TIF)

S6 Fig Complementation between fragments of cpM.SssI. Plasmids were digested with Hin6I. Lane 1, pB-Sss[1–57] Lane 2, pB-Sss[58–275] Lane 3, pOB-Sss[276–57] Lane 4, pOB-Sss[243–57] Lane 5, pOB-Sss[58–386] Lanes 6 and 7, pB-Sss[1–57] + pOB-Sss[58–386] Lanes 8 and 9, pB-Sss[58–275] + pOB-Sss[276–57] Lanes 10 and 11, pB-Sss[58–275] + pOB-Sss[243–57] M, 1 kb GeneRuler Induced cultures were grown in the presence of 0.1% arabinose at 30°C for 5 hours.
(TIF)

S7 Fig SDS-polyacrylamide gel electrophoresis of extracts prepared from *E*. *coli* cells producing circularly permuted variants of M.MpeI. S, soluble fraction; T, total extract. M, molecular weight marker. Bands corresponding to the cpM.MpeI variants are indicated by arrowhead.
(TIFF)

S8 Fig SDS-polyacrylamide gel electrophoresis of extracts prepared from *E*. *coli* cells producing circularly permuted variants of M.SssI. S, soluble fraction; T, total extract. M, molecular weight marker. Bands corresponding to the cpM.SssI variants are indicated by arrowhead.
(TIFF)

S9 Fig SDS-polyacrylamide gel electrophoresis of extracts prepared from *E*. *coli* cells producing the [192–61] or the [62–279] fragment of cp62M.MpeI. S, soluble fraction; T, total extract. M, molecular weight marker. Bands correspondig to the overproduced fragments are marked by arrowhead. The scheme under the gel shows the arrangement of the conserved motifs in the fragments. The fragments are inactive by themselves (empty boxes), but can assemble to produce a low activity enzyme when produced in the same *E*. *coli* cell (filled boxes).
(TIFF)

S10 Fig Permutation sites of the circularly permuted M.MpeI variants mapped on the M.MpeI structure. The sites yielding active and inactive MTase are highlighted in green and red, respectively. The yellow numbers indicate the positions of the N-terminal amino acids of the CP variants.
(TIFF)

S11 Fig Permutation sites of the circularly permuted M.SssI variants mapped on the predicted structure of M.SssI. The sites yielding active and inactive MTase are highlighted in green and red, respectively. The yellow numbers indicate the positions of the N-terminal amino acids of the CP variants.
(TIFF)

S12 Fig Methyltransferase activity of cpM.MpeI variants measured in cell extracts. Incorporation of [methyl-^3^H] into DNA. The radioactivity values (cpm) were normalized to culture densities (OD_600_ measured from four-fold diluted cultures). Empty bars, uninduced cultures; filled bars, arabinose-induced cultures. Results with the induced cultures are averages derived from three independent cultures. Error bars: standard error of the mean. Data with the uninduced cultures represent a single experiment. The figure was created with GraphPad Prism.
(TIF)

S13 Fig Methyltransferase activity of cpM.SssI variants measured in cell extracts of arabinose-incuced cultures. Incorporation of [methyl-^3^H] into DNA. Results are averages derived from three independent cultures. Error bars: standard error of the mean. The figure was created with GraphPad Prism.
(TIF)

S14 Fig N-termini of circularly permuted variants of M.MpeI, M.SssI and M.HaeIII. Clustal-Omega alignment.
(DOCX)

S1 Table Oligonucleotides used in this work. (DOCX)

S2 Table Plasmids expressing fragments of cpM.MpeI and cpM.SssI. (DOCX)
