# Supplementary material for: Circularly permuted variants of two CG-specific prokaryotic DNA methyltransferases
Source: PLoS One. 2018 May 10;13(5):e0197232. doi: 10.1371/journal.pone.0197232 (PMC5944983; doi:10.1371/journal.pone.0197232)
Supplement: S1 Appendix — (DOCX) [file pone.0197232.s001.docx]

**Search of the REBASE database for circularly permuted C5-MTases**

A file containing translated ORFs of characterized and putative C5-MTases (20677 entries) was downloaded from <http://rebase.neb.com/cgi-bin/seqsget?D+ap> and was processed by a script written in python (cpmtase_finder.py):

1. Load the sequence entries using the Bio.SeqIO module of the Biopython package {Cock, 2009 #78}.

2. Find motifs I and X using the regular expressions "[SED][AFILTV]F[ACDST]G[ACIMLPTVY] [GM]" and "GN[SAG]", respectively. The parameters were optimized to find all cpMTases known to have circularly permuted structure (M.Alw26I, M2.BsaI, M.BssHII, M.Esp3I and M.Eco31I), but were kept as strict as possible to exclude false positives.

3. Select the sequences in which motif X precedes motif I.

The search gave 53 results. Each sequence was checked manually to exclude false positives.
