# Supplementary material for: Circularly permuted variants of two CG-specific prokaryotic DNA methyltransferases
Source: PLoS One. 2018 May 10;13(5):e0197232. doi: 10.1371/journal.pone.0197232 (PMC5944983; doi:10.1371/journal.pone.0197232)
Supplement: S1 Dataset — (DOCX) [file pone.0197232.s002.docx]

>Tandemly duplicated M.MpeI 802 aa 93.75 kDa

MGNSNKDKIKVIKVFEAFAGIGSQFKALKNIARSKNWEIQHSGMVEWFVDAIVSYVAIHSKNFNPKIERLDRDILSISNDSKMPISEYGIKKINNTIKASYLNYAKKHFNNLFDIKKVNKDNFPKNIDIFTYSFPCQDLSVQGLQKGIDKELNTRSGLLWEIERILEEIKNSFSKEEMPKYLLMENVKNLLSHKNKKNYNTWLKQLEKFGYKSKTYLLNSKNFDNCQNRERVFCLSIRDDYLEKTGFKFKELEKVKNPPKKIKDILVDSSNYKYLNLNKYETTTFRETKSNIISRPLKNYTTFNSENYVYNINGIGPTLTASGANSRIKIETQQGVRYLTPLECFKYMQFDVNDFKKVQSTNLISENKMIYIAGNSIPVKILEAIFNTLEFVNNEELEMGNSNKDKIKVIKVFEAFAGIGSQFKALKNIARSKNWEIQHSGMVEWFVDAIVSYVAIHSKNFNPKIERLDRDILSISNDSKMPISEYGIKKINNTIKASYLNYAKKHFNNLFDIKKVNKDNFPKNIDIFTYSFPCQDLSVQGLQKGIDKELNTRSGLLWEIERILEEIKNSFSKEEMPKYLLMENVKNLLSHKNKKNYNTWLKQLEKFGYKSKTYLLNSKNFDNCQNRERVFCLSIRDDYLEKTGFKFKELEKVKNPPKKIKDILVDSSNYKYLNLNKYETTTFRETKSNIISRPLKNYTTFNSENYVYNINGIGPTLTASGANSRIKIETQQGVRYLTPLECFKYMQFDVNDFKKVQSTNLISENKMIYIAGNSIPVKILEAIFNTLEFVNNEELEHHHHHH

>cp35M.MpeI 400 aa, 46.66 kDa

MGNWEIQHSGMVEWFVDAIVSYVAIHSKNFNPKIERLDRDILSISNDSKMPISEYGIKKINNTIKASYLNYAKKHFNNLFDIKKVNKDNFPKNIDIFTYSFPCQDLSVQGLQKGIDKELNTRSGLLWEIERILEEIKNSFSKEEMPKYLLMENVKNLLSHKNKKNYNTWLKQLEKFGYKSKTYLLNSKNFDNCQNRERVFCLSIRDDYLEKTGFKFKELEKVKNPPKKIKDILVDSSNYKYLNLNKYETTTFRETKSNIISRPLKNYTTFNSENYVYNINGIGPTLTASGANSRIKIETQQGVRYLTPLECFKYMQFDVNDFKKVQSTNLISENKMIYIAGNSIPVKILEAIFNTLEFVNNEELEMGNSNKDKIKVIKVFEAFAGIGSQFKALKNIARSK

>cp62M.MpeI 400 aa, 46.66 kDa

MGFNPKIERLDRDILSISNDSKMPISEYGIKKINNTIKASYLNYAKKHFNNLFDIKKVNKDNFPKNIDIFTYSFPCQDLSVQGLQKGIDKELNTRSGLLWEIERILEEIKNSFSKEEMPKYLLMENVKNLLSHKNKKNYNTWLKQLEKFGYKSKTYLLNSKNFDNCQNRERVFCLSIRDDYLEKTGFKFKELEKVKNPPKKIKDILVDSSNYKYLNLNKYETTTFRETKSNIISRPLKNYTTFNSENYVYNINGIGPTLTASGANSRIKIETQQGVRYLTPLECFKYMQFDVNDFKKVQSTNLISENKMIYIAGNSIPVKILEAIFNTLEFVNNEELEMGNSNKDKIKVIKVFEAFAGIGSQFKALKNIARSKNWEIQHSGMVEWFVDAIVSYVAIHSKN

>cp122M.MpeI 400 aa, 46.66 kDa

MGFPKNIDIFTYSFPCQDLSVQGLQKGIDKELNTRSGLLWEIERILEEIKNSFSKEEMPKYLLMENVKNLLSHKNKKNYNTWLKQLEKFGYKSKTYLLNSKNFDNCQNRERVFCLSIRDDYLEKTGFKFKELEKVKNPPKKIKDILVDSSNYKYLNLNKYETTTFRETKSNIISRPLKNYTTFNSENYVYNINGIGPTLTASGANSRIKIETQQGVRYLTPLECFKYMQFDVNDFKKVQSTNLISENKMIYIAGNSIPVKILEAIFNTLEFVNNEELEMGNSNKDKIKVIKVFEAFAGIGSQFKALKNIARSKNWEIQHSGMVEWFVDAIVSYVAIHSKNFNPKIERLDRDILSISNDSKMPISEYGIKKINNTIKASYLNYAKKHFNNLFDIKKVNKDN

>cp192M.MpeI 400 aa, 46.66 kDa

MGHKNKKNYNTWLKQLEKFGYKSKTYLLNSKNFDNCQNRERVFCLSIRDDYLEKTGFKFKELEKVKNPPKKIKDILVDSSNYKYLNLNKYETTTFRETKSNIISRPLKNYTTFNSENYVYNINGIGPTLTASGANSRIKIETQQGVRYLTPLECFKYMQFDVNDFKKVQSTNLISENKMIYIAGNSIPVKILEAIFNTLEFVNNEELEMGNSNKDKIKVIKVFEAFAGIGSQFKALKNIARSKNWEIQHSGMVEWFVDAIVSYVAIHSKNFNPKIERLDRDILSISNDSKMPISEYGIKKINNTIKASYLNYAKKHFNNLFDIKKVNKDNFPKNIDIFTYSFPCQDLSVQGLQKGIDKELNTRSGLLWEIERILEEIKNSFSKEEMPKYLLMENVKNLLS

>cp208M.MpeI 400 aa, 46.66 kDa

MGFGYKSKTYLLNSKNFDNCQNRERVFCLSIRDDYLEKTGFKFKELEKVKNPPKKIKDILVDSSNYKYLNLNKYETTTFRETKSNIISRPLKNYTTFNSENYVYNINGIGPTLTASGANSRIKIETQQGVRYLTPLECFKYMQFDVNDFKKVQSTNLISENKMIYIAGNSIPVKILEAIFNTLEFVNNEELEMGNSNKDKIKVIKVFEAFAGIGSQFKALKNIARSKNWEIQHSGMVEWFVDAIVSYVAIHSKNFNPKIERLDRDILSISNDSKMPISEYGIKKINNTIKASYLNYAKKHFNNLFDIKKVNKDNFPKNIDIFTYSFPCQDLSVQGLQKGIDKELNTRSGLLWEIERILEEIKNSFSKEEMPKYLLMENVKNLLSHKNKKNYNTWLKQLEK

>cp215M.MpeI 400 aa, 46.66 kDa

MGYLLNSKNFDNCQNRERVFCLSIRDDYLEKTGFKFKELEKVKNPPKKIKDILVDSSNYKYLNLNKYETTTFRETKSNIISRPLKNYTTFNSENYVYNINGIGPTLTASGANSRIKIETQQGVRYLTPLECFKYMQFDVNDFKKVQSTNLISENKMIYIAGNSIPVKILEAIFNTLEFVNNEELEMGNSNKDKIKVIKVFEAFAGIGSQFKALKNIARSKNWEIQHSGMVEWFVDAIVSYVAIHSKNFNPKIERLDRDILSISNDSKMPISEYGIKKINNTIKASYLNYAKKHFNNLFDIKKVNKDNFPKNIDIFTYSFPCQDLSVQGLQKGIDKELNTRSGLLWEIERILEEIKNSFSKEEMPKYLLMENVKNLLSHKNKKNYNTWLKQLEKFGYKSKT

>cp222M.MpeI 400 aa, 46.66 kDa

MGYLLNSKNFDNCQNRERVFCLSIRDDYLEKTGFKFKELEKVKNPPKKIKDILVDSSNYKYLNLNKYETTTFRETKSNIISRPLKNYTTFNSENYVYNINGIGPTLTASGANSRIKIETQQGVRYLTPLECFKYMQFDVNDFKKVQSTNLISENKMIYIAGNSIPVKILEAIFNTLEFVNNEELEMGNSNKDKIKVIKVFEAFAGIGSQFKALKNIARSKNWEIQHSGMVEWFVDAIVSYVAIHSKNFNPKIERLDRDILSISNDSKMPISEYGIKKINNTIKASYLNYAKKHFNNLFDIKKVNKDNFPKNIDIFTYSFPCQDLSVQGLQKGIDKELNTRSGLLWEIERILEEIKNSFSKEEMPKYLLMENVKNLLSHKNKKNYNTWLKQLEKFGYKSKT

>cp245M.MpeI 399 aa, 46.66 kDa

MGFKFKELEKVKNPPKKIKDILVDSSNYKYLNLNKYETTTFRETKSNIISRPLKNYTTFNSENYVYNINGIGPTLTASGANSRIKIETQQGVRYLTPLECFKYMQFDVNDFKKVQSTNLISENKMIYIAGNSIPVKILEAIFNTLEFVNNEELEMGNSNKDKIKVIKVFEAFAGIGSQFKALKNIARSKNWEIQHSGMVEWFVDAIVSYVAIHSKNFNPKIERLDRDILSISNDSKMPISEYGIKKINNTIKASYLNYAKKHFNNLFDIKKVNKDNFPKNIDIFTYSFPCQDLSVQGLQKGIDKELNTRSGLLWEIERILEEIKNSFSKEEMPKYLLMENVKNLLSHKNKKNYNTWLKQLEKFGYKSKTYLLNSKNFDNCQNRERVFCLSIRDDYLEKT

 >cp280M.MpeI 399 aa, 46.66 kDa

METTTFRETKSNIISRPLKNYTTFNSENYVYNINGIGPTLTASGANSRIKIETQQGVRYLTPLECFKYMQFDVNDFKKVQSTNLISENKMIYIAGNSIPVKILEAIFNTLEFVNNEELEMGNSNKDKIKVIKVFEAFAGIGSQFKALKNIARSKNWEIQHSGMVEWFVDAIVSYVAIHSKNFNPKIERLDRDILSISNDSKMPISEYGIKKINNTIKASYLNYAKKHFNNLFDIKKVNKDNFPKNIDIFTYSFPCQDLSVQGLQKGIDKELNTRSGLLWEIERILEEIKNSFSKEEMPKYLLMENVKNLLSHKNKKNYNTWLKQLEKFGYKSKTYLLNSKNFDNCQNRERVFCLSIRDDYLEKTGFKFKELEKVKNPPKKIKDILVDSSNYKYLNLNKY

>cp332M.MpeI 400 aa, 46.66 kDa

MGQQGVRYLTPLECFKYMQFDVNDFKKVQSTNLISENKMIYIAGNSIPVKILEAIFNTLEFVNNEELEMGNSNKDKIKVIKVFEAFAGIGSQFKALKNIARSKNWEIQHSGMVEWFVDAIVSYVAIHSKNFNPKIERLDRDILSISNDSKMPISEYGIKKINNTIKASYLNYAKKHFNNLFDIKKVNKDNFPKNIDIFTYSFPCQDLSVQGLQKGIDKELNTRSGLLWEIERILEEIKNSFSKEEMPKYLLMENVKNLLSHKNKKNYNTWLKQLEKFGYKSKTYLLNSKNFDNCQNRERVFCLSIRDDYLEKTGFKFKELEKVKNPPKKIKDILVDSSNYKYLNLNKYETTTFRETKSNIISRPLKNYTTFNSENYVYNINGIGPTLTASGANSRIKIET

>cp351M.MpeI 399 aa, 46.66 kDa

MVNDFKKVQSTNLISENKMIYIAGNSIPVKILEAIFNTLEFVNNEELEMGNSNKDKIKVIKVFEAFAGIGSQFKALKNIARSKNWEIQHSGMVEWFVDAIVSYVAIHSKNFNPKIERLDRDILSISNDSKMPISEYGIKKINNTIKASYLNYAKKHFNNLFDIKKVNKDNFPKNIDIFTYSFPCQDLSVQGLQKGIDKELNTRSGLLWEIERILEEIKNSFSKEEMPKYLLMENVKNLLSHKNKKNYNTWLKQLEKFGYKSKTYLLNSKNFDNCQNRERVFCLSIRDDYLEKTGFKFKELEKVKNPPKKIKDILVDSSNYKYLNLNKYETTTFRETKSNIISRPLKNYTTFNSENYVYNINGIGPTLTASGANSRIKIETQQGVRYLTPLECFKYMQFD

>cp357M.MpeI 399 aa, 46.66 kDa

MVQSTNLISENKMIYIAGNSIPVKILEAIFNTLEFVNNEELEMGNSNKDKIKVIKVFEAFAGIGSQFKALKNIARSKNWEIQHSGMVEWFVDAIVSYVAIHSKNFNPKIERLDRDILSISNDSKMPISEYGIKKINNTIKASYLNYAKKHFNNLFDIKKVNKDNFPKNIDIFTYSFPCQDLSVQGLQKGIDKELNTRSGLLWEIERILEEIKNSFSKEEMPKYLLMENVKNLLSHKNKKNYNTWLKQLEKFGYKSKTYLLNSKNFDNCQNRERVFCLSIRDDYLEKTGFKFKELEKVKNPPKKIKDILVDSSNYKYLNLNKYETTTFRETKSNIISRPLKNYTTFNSENYVYNINGIGPTLTASGANSRIKIETQQGVRYLTPLECFKYMQFDVNDFKK

>cp361M.MpeI 400 aa, 46.66 kDa

MGNLISENKMIYIAGNSIPVKILEAIFNTLEFVNNEELEMGNSNKDKIKVIKVFEAFAGIGSQFKALKNIARSKNWEIQHSGMVEWFVDAIVSYVAIHSKNFNPKIERLDRDILSISNDSKMPISEYGIKKINNTIKASYLNYAKKHFNNLFDIKKVNKDNFPKNIDIFTYSFPCQDLSVQGLQKGIDKELNTRSGLLWEIERILEEIKNSFSKEEMPKYLLMENVKNLLSHKNKKNYNTWLKQLEKFGYKSKTYLLNSKNFDNCQNRERVFCLSIRDDYLEKTGFKFKELEKVKNPPKKIKDILVDSSNYKYLNLNKYETTTFRETKSNIISRPLKNYTTFNSENYVYNINGIGPTLTASGANSRIKIETQQGVRYLTPLECFKYMQFDVNDFKKVQST

>cp377M.MpeI 399 aa, 46.6 kDa

MVKILEAIFNTLEFVNNEELEMGNSNKDKIKVIKVFEAFAGIGSQFKALKNIARSKNWEIQHSGMVEWFVDAIVSYVAIHSKNFNPKIERLDRDILSISNDSKMPISEYGIKKINNTIKASYLNYAKKHFNNLFDIKKVNKDNFPKNIDIFTYSFPCQDLSVQGLQKGIDKELNTRSGLLWEIERILEEIKNSFSKEEMPKYLLMENVKNLLSHKNKKNYNTWLKQLEKFGYKSKTYLLNSKNFDNCQNRERVFCLSIRDDYLEKTGFKFKELEKVKNPPKKIKDILVDSSNYKYLNLNKYETTTFRETKSNIISRPLKNYTTFNSENYVYNINGIGPTLTASGANSRIKIETQQGVRYLTPLECFKYMQFDVNDFKKVQSTNLISENKMIYIAGNSIP

>tdM.MpeI-280 (with GGGSGLE linker) 809 aa, 94.3 kDa

MGNSNKDKIKVIKVFEAFAGIGSQFKALKNIARSKNWEIQHSGMVEWFVDAIVSYVAIHSKNFNPKIERLDRDILSISNDSKMPISEYGIKKINNTIKASYLNYAKKHFNNLFDIKKVNKDNFPKNIDIFTYSFPCQDLSVQGLQKGIDKELNTRSGLLWEIERILEEIKNSFSKEEMPKYLLMENVKNLLSHKNKKNYNTWLKQLEKFGYKSKTYLLNSKNFDNCQNRERVFCLSIRDDYLEKTGFKFKELEKVKNPPKKIKDILVDSSNYKYLNLNKYETTTFRETKSNIISRPLKNYTTFNSENYVYNINGIGPTLTASGANSRIKIETQQGVRYLTPLECFKYMQFDVNDFKKVQSTNLISENKMIYIAGNSIPVKILEAIFNTLEFVNNEELEGGGSGLEMGNSNKDKIKVIKVFEAFAGIGSQFKALKNIARSKNWEIQHSGMVEWFVDAIVSYVAIHSKNFNPKIERLDRDILSISNDSKMPISEYGIKKINNTIKASYLNYAKKHFNNLFDIKKVNKDNFPKNIDIFTYSFPCQDLSVQGLQKGIDKELNTRSGLLWEIERILEEIKNSFSKEEMPKYLLMENVKNLLSHKNKKNYNTWLKQLEKFGYKSKTYLLNSKNFDNCQNRERVFCLSIRDDYLEKTGFKFKELEKVKNPPKKIKDILVDSSNYKYLNLNKYETTTFRETKSNIISRPLKNYTTFNSENYVYNINGIGPTLTASGANSRIKIETQQGVRYLTPLECFKYMQFDVNDFKKVQSTNLISENKMIYIAGNSIPVKILEAIFNTLEFVNNEELEHHHHHH

>cp62M.MpeI-280 (with GGGSGLE linker) 407 aa, 47.22 kDa

MGFNPKIERLDRDILSISNDSKMPISEYGIKKINNTIKASYLNYAKKHFNNLFDIKKVNKDNFPKNIDIFTYSFPCQDLSVQGLQKGIDKELNTRSGLLWEIERILEEIKNSFSKEEMPKYLLMENVKNLLSHKNKKNYNTWLKQLEKFGYKSKTYLLNSKNFDNCQNRERVFCLSIRDDYLEKTGFKFKELEKVKNPPKKIKDILVDSSNYKYLNLNKYETTTFRETKSNIISRPLKNYTTFNSENYVYNINGIGPTLTASGANSRIKIETQQGVRYLTPLECFKYMQFDVNDFKKVQSTNLISENKMIYIAGNSIPVKILEAIFNTLEFVNNEELEGGGSGLEMGNSNKDKIKVIKVFEAFAGIGSQFKALKNIARSKNWEIQHSGMVEWFVDAIVSYVAIHSKN
