# Supplementary material for: Circularly permuted variants of two CG-specific prokaryotic DNA methyltransferases
Source: PLoS One. 2018 May 10;13(5):e0197232. doi: 10.1371/journal.pone.0197232 (PMC5944983; doi:10.1371/journal.pone.0197232)
Supplement: S2 Dataset — (DOCX) [file pone.0197232.s003.docx]

>Tandemly duplicated M.SssI 775 aa, 88.99 kDa

MSKVENKTKKLRVFEAFAGIGAQRKALEKVRKDEYEIVGLAEWYVPAIVMYQAIHNNFHTKLEYKSVSREEMIDYLENKTLSWNSKNPVSNGYWKRKKDDELKIIYNAIKLSEKEGNIFDIRDLYKRTLKNIDLLTYSFPCQDLSQQGIQKGMKRGSGTRSGLLWEIERALDSTEKNDLPKYLLMENVGALLHKKNEEELNQWKQKLESLGYQNSIEVLNAADFGSSQARRRVFMISTLNEFVELPKGDKKPKSIKKVLNKIVSEKDILNNLLKYNLTEFKKTKSNINKASLIGYSKFNSEGYVYDPEFTGPTLTASGANSRIKIKDGSNIRKMNSDETFLYMGFDSQDGKRVNEIEFLTENQKIFVCGNSISVEVLEAIIDKIGGLECMSKVENKTKKLRVFEAFAGIGAQRKALEKVRKDEYEIVGLAEWYVPAIVMYQAIHNNFHTKLEYKSVSREEMIDYLENKTLSWNSKNPVSNGYWKRKKDDELKIIYNAIKLSEKEGNIFDIRDLYKRTLKNIDLLTYSFPCQDLSQQGIQKGMKRGSGTRSGLLWEIERALDSTEKNDLPKYLLMENVGALLHKKNEEELNQWKQKLESLGYQNSIEVLNAADFGSSQARRRVFMISTLNEFVELPKGDKKPKSIKKVLNKIVSEKDILNNLLKYNLTEFKKTKSNINKASLIGYSKFNSEGYVYDPEFTGPTLTASGANSRIKIKDGSNIRKMNSDETFLYMGFDSQDGKRVNEIEFLTENQKIFVCGNSISVEVLEAIIDKIGG

>cp33M.SssI 390 aa, 44.8 kDa

MDEYEIVGLAEWYVPAIVMYQAIHNNFHTKLEYKSVSREEMIDYLENKTLSWNSKNPVSNGYWKRKKDDELKIIYNAIKLSEKEGNIFDIRDLYKRTLKNIDLLTYSFPCQDLSQQGIQKGMKRGSGTRSGLLWEIERALDSTEKNDLPKYLLMENVGALLHKKNEEELNQWKQKLESLGYQNSIEVLNAADFGSSQARRRVFMISTLNEFVELPKGDKKPKSIKKVLNKIVSEKDILNNLLKYNLTEFKKTKSNINKASLIGYSKFNSEGYVYDPEFTGPTLTASGANSRIKIKDGSNIRKMNSDETFLYMGFDSQDGKRVNEIEFLTENQKIFVCGNSISVEVLEAIIDKIGGLECMSKVENKTKKLRVFEAFAGIGAQRKALEKVRK

>cp58M.SssI 391 aa, 44.86 kDa

MGFHTKLEYKSVSREEMIDYLENKTLSWNSKNPVSNGYWKRKKDDELKIIYNAIKLSEKEGNIFDIRDLYKRTLKNIDLLTYSFPCQDLSQQGIQKGMKRGSGTRSGLLWEIERALDSTEKNDLPKYLLMENVGALLHKKNEEELNQWKQKLESLGYQNSIEVLNAADFGSSQARRRVFMISTLNEFVELPKGDKKPKSIKKVLNKIVSEKDILNNLLKYNLTEFKKTKSNINKASLIGYSKFNSEGYVYDPEFTGPTLTASGANSRIKIKDGSNIRKMNSDETFLYMGFDSQDGKRVNEIEFLTENQKIFVCGNSISVEVLEAIIDKIGGLECMSKVENKTKKLRVFEAFAGIGAQRKALEKVRKDEYEIVGLAEWYVPAIVMYQAIHNN

>cp156M.SssI 391 aa, 44.86 kDa

MGSGTRSGLLWEIERALDSTEKNDLPKYLLMENVGALLHKKNEEELNQWKQKLESLGYQNSIEVLNAADFGSSQARRRVFMISTLNEFVELPKGDKKPKSIKKVLNKIVSEKDILNNLLKYNLTEFKKTKSNINKASLIGYSKFNSEGYVYDPEFTGPTLTASGANSRIKIKDGSNIRKMNSDETFLYMGFDSQDGKRVNEIEFLTENQKIFVCGNSISVEVLEAIIDKIGGLECMSKVENKTKKLRVFEAFAGIGAQRKALEKVRKDEYEIVGLAEWYVPAIVMYQAIHNNFHTKLEYKSVSREEMIDYLENKTLSWNSKNPVSNGYWKRKKDDELKIIYNAIKLSEKEGNIFDIRDLYKRTLKNIDLLTYSFPCQDLSQQGIQKGMKRG

>cp173M.SssI 391 aa, 44.86 kDa

MGSTEKNDLPKYLLMENVGALLHKKNEEELNQWKQKLESLGYQNSIEVLNAADFGSSQARRRVFMISTLNEFVELPKGDKKPKSIKKVLNKIVSEKDILNNLLKYNLTEFKKTKSNINKASLIGYSKFNSEGYVYDPEFTGPTLTASGANSRIKIKDGSNIRKMNSDETFLYMGFDSQDGKRVNEIEFLTENQKIFVCGNSISVEVLEAIIDKIGGLECMSKVENKTKKLRVFEAFAGIGAQRKALEKVRKDEYEIVGLAEWYVPAIVMYQAIHNNFHTKLEYKSVSREEMIDYLENKTLSWNSKNPVSNGYWKRKKDDELKIIYNAIKLSEKEGNIFDIRDLYKRTLKNIDLLTYSFPCQDLSQQGIQKGMKRGSGTRSGLLWEIERALD

>cp243M.SssI 390 aa, 44.8 kDa

MVELPKGDKKPKSIKKVLNKIVSEKDILNNLLKYNLTEFKKTKSNINKASLIGYSKFNSEGYVYDPEFTGPTLTASGANSRIKIKDGSNIRKMNSDETFLYMGFDSQDGKRVNEIEFLTENQKIFVCGNSISVEVLEAIIDKIGGLECMSKVENKTKKLRVFEAFAGIGAQRKALEKVRKDEYEIVGLAEWYVPAIVMYQAIHNNFHTKLEYKSVSREEMIDYLENKTLSWNSKNPVSNGYWKRKKDDELKIIYNAIKLSEKEGNIFDIRDLYKRTLKNIDLLTYSFPCQDLSQQGIQKGMKRGSGTRSGLLWEIERALDSTEKNDLPKYLLMENVGALLHKKNEEELNQWKQKLESLGYQNSIEVLNAADFGSSQARRRVFMISTLNEF

>cp308M.SssI 390 aa, 44.8 kDa

MEFTGPTLTASGANSRIKIKDGSNIRKMNSDETFLYMGFDSQDGKRVNEIEFLTENQKIFVCGNSISVEVLEAIIDKIGGLECMSKVENKTKKLRVFEAFAGIGAQRKALEKVRKDEYEIVGLAEWYVPAIVMYQAIHNNFHTKLEYKSVSREEMIDYLENKTLSWNSKNPVSNGYWKRKKDDELKIIYNAIKLSEKEGNIFDIRDLYKRTLKNIDLLTYSFPCQDLSQQGIQKGMKRGSGTRSGLLWEIERALDSTEKNDLPKYLLMENVGALLHKKNEEELNQWKQKLESLGYQNSIEVLNAADFGSSQARRRVFMISTLNEFVELPKGDKKPKSIKKVLNKIVSEKDILNNLLKYNLTEFKKTKSNINKASLIGYSKFNSEGYVYDP

>cp357M.SssI 390 aa, 44.8 kDa

MEFLTENQKIFVCGNSISVEVLEAIIDKIGGLECMSKVENKTKKLRVFEAFAGIGAQRKALEKVRKDEYEIVGLAEWYVPAIVMYQAIHNNFHTKLEYKSVSREEMIDYLENKTLSWNSKNPVSNGYWKRKKDDELKIIYNAIKLSEKEGNIFDIRDLYKRTLKNIDLLTYSFPCQDLSQQGIQKGMKRGSGTRSGLLWEIERALDSTEKNDLPKYLLMENVGALLHKKNEEELNQWKQKLESLGYQNSIEVLNAADFGSSQARRRVFMISTLNEFVELPKGDKKPKSIKKVLNKIVSEKDILNNLLKYNLTEFKKTKSNINKASLIGYSKFNSEGYVYDPEFTGPTLTASGANSRIKIKDGSNIRKMNSDETFLYMGFDSQDGKRVNEI

>cp58M.SssI-280 (with LEGGGSGLEC-linker) 398 aa, 45.42 kDa

MGFHTKLEYKSVSREEMIDYLENKTLSWNSKNPVSNGYWKRKKDDELKIIYNAIKLSEKEGNIFDIRDLYKRTLKNIDLLTYSFPCQDLSQQGIQKGMKRGSGTRSGLLWEIERALDSTEKNDLPKYLLMENVGALLHKKNEEELNQWKQKLESLGYQNSIEVLNAADFGSSQARRRVFMISTLNEFVELPKGDKKPKSIKKVLNKIVSEKDILNNLLKYNLTEFKKTKSNINKASLIGYSKFNSEGYVYDPEFTGPTLTASGANSRIKIKDGSNIRKMNSDETFLYMGFDSQDGKRVNEIEFLTENQKIFVCGNSISVEVLEAIIDKIGGLEGGGSGLECMSKVENKTKKLRVFEAFAGIGAQRKALEKVRKDEYEIVGLAEWYVPAIVMYQAIHNN
