# Supplementary material for: Circularly permuted variants of two CG-specific prokaryotic DNA methyltransferases
Source: PLoS One. 2018 May 10;13(5):e0197232. doi: 10.1371/journal.pone.0197232 (PMC5944983; doi:10.1371/journal.pone.0197232)
Supplement: S3 Dataset — (DOCX) [file pone.0197232.s004.docx]

> Mpe[1-61] 62 aa 6.98 kDa

MGNSNKDKIKVIKVFEAFAGIGSQFKALKNIARSKNWEIQHSGMVEWFVDAIVSYVAIHSKN

> Mpe[62-395] 338 aa 39.7 kDa

MGFNPKIERLDRDILSISNDSKMPISEYGIKKINNTIKASYLNYAKKHFNNLFDIKKVNKDNFPKNIDIFTYSFPCQDLSVQGLQKGIDKELNTRSGLLWEIERILEEIKNSFSKEEMPKYLLMENVKNLLSHKNKKNYNTWLKQLEKFGYKSKTYLLNSKNFDNCQNRERVFCLSIRDDYLEKTGFKFKELEKVKNPPKKIKDILVDSSNYKYLNLNKYETTTFRETKSNIISRPLKNYTTFNSENYVYNINGIGPTLTASGANSRIKIETQQGVRYLTPLECFKYMQFDVNDFKKVQSTNLISENKMIYIAGNSIPVKILEAIFNTLEFVNNEELE

>Mpe[192-61] 270 aa, 31.41 kDa

MGHKNKKNYNTWLKQLEKFGYKSKTYLLNSKNFDNCQNRERVFCLSIRDDYLEKTGFKFKELEKVKNPPKKIKDILVDSSNYKYLNLNKYETTTFRETKSNIISRPLKNYTTFNSENYVYNINGIGPTLTASGANSRIKIETQQGVRYLTPLECFKYMQFDVNDFKKVQSTNLISENKMIYIAGNSIPVKILEAIFNTLEFVNNEELEMGNSNKDKIKVIKVFEAFAGIGSQFKALKNIARSKNWEIQHSGMVEWFVDAIVSYVAIHSKN

>Mpe[62-191] 132 aa, 15.45 kDa

MGFNPKIERLDRDILSISNDSKMPISEYGIKKINNTIKASYLNYAKKHFNNLFDIKKVNKDNFPKNIDIFTYSFPCQDLSVQGLQKGIDKELNTRSGLLWEIERILEEIKNSFSKEEMPKYLLMENVKNLLS

>Mpe[361-244] 284 aa, 33.17 kDa

MGNLISENKMIYIAGNSIPVKILEAIFNTLEFVNNEELEMGNSNKDKIKVIKVFEAFAGIGSQFKALKNIARSKNWEIQHSGMVEWFVDAIVSYVAIHSKNFNPKIERLDRDILSISNDSKMPISEYGIKKINNTIKASYLNYAKKHFNNLFDIKKVNKDNFPKNIDIFTYSFPCQDLSVQGLQKGIDKELNTRSGLLWEIERILEEIKNSFSKEEMPKYLLMENVKNLLSHKNKKNYNTWLKQLEKFGYKSKTYLLNSKNFDNCQNRERVFCLSIRDDYLEKT

>Mpe[245-360] 117 aa, 13.64 kDa

MGFKFKELEKVKNPPKKIKDILVDSSNYKYLNLNKYETTTFRETKSNIISRPLKNYTTFNSENYVYNINGIGPTLTASGANSRIKIETQQGVRYLTPLECFKYMQFDVNDFKKVQST

>Mpe[280-61] 181 aa, 20.62 kDa

METTTFRETKSNIISRPLKNYTTFNSENYVYNINGIGPTLTASGANSRIKIETQQGVRYLTPLECFKYMQFDVNDFKKVQSTNLISENKMIYIAGNSIPVKILEAIFNTLEFVNNEELEMGNSNKDKIKVIKVFEAFAGIGSQFKALKNIARSKNWEIQHSGMVEWFVDAIVSYVAIHSKN

>Mpe[62-279] 220 aa, 26.18 kDa

MGFNPKIERLDRDILSISNDSKMPISEYGIKKINNTIKASYLNYAKKHFNNLFDIKKVNKDNFPKNIDIFTYSFPCQDLSVQGLQKGIDKELNTRSGLLWEIERILEEIKNSFSKEEMPKYLLMENVKNLLSHKNKKNYNTWLKQLEKFGYKSKTYLLNSKNFDNCQNRERVFCLSIRDDYLEKTGFKFKELEKVKNPPKKIKDILVDSSNYKYLNLNKY
