# Supplementary material for: Circularly permuted variants of two CG-specific prokaryotic DNA methyltransferases
Source: PLoS One. 2018 May 10;13(5):e0197232. doi: 10.1371/journal.pone.0197232 (PMC5944983; doi:10.1371/journal.pone.0197232)
Supplement: S4 Dataset — (DOCX) [file pone.0197232.s005.docx]

> Sss[1-57] 57 aa 6.58 kDa

MSKVENKTKKLRVFEAFAGIGAQRKALEKVRKDEYEIVGLAEWYVPAIVMYQAIHNN

> Sss[58-386] 332 aa 37.77 kDa

FHTKLEYKSVSREEMIDYLENKTLSWNSKNPVSNGYWKRKKDDELKIIYNAIKLSEKEGNIFDIRDLYKRTLKNIDLLTYSFPCQDLSQQGIQKGMKRGSGTRSGLLWEIERALDSTEKNDLPKYLLMENVGALLHKKNEEELNQWKQKLESLGYQNSIEVLNAADFGSSQARRRVFMISTLNEFVELPKGDKKPKSIKKVLNKIVSEKDILNNLLKYNLTEFKKTKSNINKASLIGYSKFNSEGYVYDPEFTGPTLTASGANSRIKIKDGSNIRKMNSDETFLYMGFDSQDGKRVNEIEFLTENQKIFVCGNSISVEVLEAIIDKIGG

>Sss[58-242] 187 aa, 21.87 kDa

MGFHTKLEYKSVSREEMIDYLENKTLSWNSKNPVSNGYWKRKKDDELKIIYNAIKLSEKEGNIFDIRDLYKRTLKNIDLLTYSFPCQDLSQQGIQKGMKRGSGTRSGLLWEIERALDSTEKNDLPKYLLMENVGALLHKKNEEELNQWKQKLESLGYQNSIEVLNAADFGSSQARRRVFMISTLNEF

>Sss[58-275] 220 aa, 25.64 kDa

MGFHTKLEYKSVSREEMIDYLENKTLSWNSKNPVSNGYWKRKKDDELKIIYNAIKLSEKEGNIFDIRDLYKRTLKNIDLLTYSFPCQDLSQQGIQKGMKRGSGTRSGLLWEIERALDSTEKNDLPKYLLMENVGALLHKKNEEELNQWKQKLESLGYQNSIEVLNAADFGSSQARRRVFMISTLNEFVELPKGDKKPKSIKKVLNKIVSEKDILNNLLKY

>Sss[276-57] 173 aa, 19.43 kDa

MGNLTEFKKTKSNINKASLIGYSKFNSEGYVYDPEFTGPTLTASGANSRIKIKDGSNIRKMNSDETFLYMGFDSQDGKRVNEIEFLTENQKIFVCGNSISVEVLEAIIDKIGGLECMSKVENKTKKLRVFEAFAGIGAQRKALEKVRKDEYEIVGLAEWYVPAIVMYQAIHNN

>Sss[357-242] 276 aa, 31.97 kDa

MEFLTENQKIFVCGNSISVEVLEAIIDKIGGLECMSKVENKTKKLRVFEAFAGIGAQRKALEKVRKDEYEIVGLAEWYVPAIVMYQAIHNNFHTKLEYKSVSREEMIDYLENKTLSWNSKNPVSNGYWKRKKDDELKIIYNAIKLSEKEGNIFDIRDLYKRTLKNIDLLTYSFPCQDLSQQGIQKGMKRGSGTRSGLLWEIERALDSTEKNDLPKYLLMENVGALLHKKNEEELNQWKQKLESLGYQNSIEVLNAADFGSSQARRRVFMISTLNEF

>Sss[243-57] 205 aa, 23.18 kDa

MVELPKGDKKPKSIKKVLNKIVSEKDILNNLLKYNLTEFKKTKSNINKASLIGYSKFNSEGYVYDPEFTGPTLTASGANSRIKIKDGSNIRKMNSDETFLYMGFDSQDGKRVNEIEFLTENQKIFVCGNSISVEVLEAIIDKIGGLECMSKVENKTKKLRVFEAFAGIGAQRKALEKVRKDEYEIVGLAEWYVPAIVMYQAIHNN

>Sss[243-356] 115 aa, 12.99 kDa

MVELPKGDKKPKSIKKVLNKIVSEKDILNNLLKYNLTEFKKTKSNINKASLIGYSKFNSEGYVYDPEFTGPTLTASGANSRIKIKDGSNIRKMNSDETFLYMGFDSQDGKRVNEI
