# Supplementary material for: Circularly permuted variants of two CG-specific prokaryotic DNA methyltransferases
Source: PLoS One. 2018 May 10;13(5):e0197232. doi: 10.1371/journal.pone.0197232 (PMC5944983; doi:10.1371/journal.pone.0197232)
Supplement: S5 Dataset — (DOCX) [file pone.0197232.s006.docx]

M.Bli388ORF2814P SDRLHTSSLNSPIVVERIRHLPVGGRMKDIPPHLWHDS---Y--VRVGEKKTGGPNLRLL 59

M.HauORF2644P KITNHYTRNVGMQVQERINHLGIGQKMQDLPEHLWHESFRYY--VKEDPSRQGGPNLRMI 60

M.BssHII FVFNHVFRRSGSEVQKRIDALKIGQKMQDLPEELWHDSFRYY--VKEDPNRRGGPNMRMI 73

M.Tbo1301ORF214495P IIENHIARRVGSDVQKRINALKIGQKMQDLPEELWHDSFKYY--VKHDPNRQGGPNLRMI 73

M.Afl2012ORF16135P VISNHIARRVGVDVQKRITALKIGQKMQDLPEELWHESFKFY--VKHDPNRQGGPNLRMI 73

M.Ski73ORF33040P VVYNHVARRVGVDVQKRINTLKIGQKMQDLPEELWHESFKFY--VKNDPNRQGGPNLRMI 73

M.EsaSS471P ------------------------------------------------------------ 0

M.Alw26I ILINNMGFKLSDLDLEMISHIPPGGNWQNISETTMKKSQRLMQIAKS-----GGRTTLYG 643

M.Ato52ORF13820P ILINNMGFKLSDLDLEMISHIPPGGNWQNISETTMKKSQRLMQIAKS-----GGRTTLYG 639

M.BsmAI EILNHTTFKLSDLDLEMIRSVPPGGNWKDIPIETVKKFKRLMRITET-----GGRTTLYG 690

M1.Hpy655ORF350P -MLNHTTSKLSDLDIEMIETVPQGGNWKHIRQETRQKSKRLQKIAQT-----GGRTTLYG 54

M.Hpy173ORF5925P ------------------------------------------------------------ 0

M1.Hpy328ORF5285P YILNHTTSKLSDLDIEMIETVPQGGNWKHIRQETRQKSKRLQKIAQT-----GGRTTLYG 117

M2.BcoDI KLYNHVVFKMGKHEAEIAPHIPEGGNWQDIPLSI--SDTRLDKIRET-----GGRTTYYG 54

M1.Bba133ORF575P KLYNHIAPRLSELEWEMAKHIPEGGNWQNIPDTI--PSQRLKQIRLS-----GGRTTYYG 56

M2.Wsppc19ORF840P KLYNHISPRLSDLEWEMAKHIPPGGNWQNIPVHL--PSKRLEQIRRT-----GGRTTYYG 54

M2.CstDORF413P MLDNHIAPSLSELDMMGISYVPEGGNWKNIPESV--PSKRLDQIRRSYAEGKGSRSTYYG 58

M2.AfaJQ135ORF6300P RIENHYAANLSELDLRICRSVPPGGNWKDIPEDI--PSERIKNIRISFAKGEGSRSTYYG 76

M.HseOs45ORFCP RIENHYAATLSELDLRICRSVPPGGNWKDIPEDI--PSERIKNIRISFAKGEGSRSTYYG 618

M2.BsaI --------------MLIIKHVPPGGNWKDIPEWV--PSKRLEQIRKSYAEGKGSRSTYYG 44

M1.RxyORF2232P MIPNHYTASLSALDLTIAQAVPPGGNWKDIPETV--PSKRLQQIRASYAAGKGSRSTYYG 66

M2.Eco31I MIPNHKAAKLSELDMMIVNSVPPGGNWKNIPLDV--PSKRIEQIRDSYAQGKGSRSTYYG 65

M1.AadSW13ORF4345P MIPNHKCSKLSDLDIKIVESVPEGGNWKNIPLNI--PSKRLEQIRESFAQGKGSRSTYYG 58

M.Ssp126ORF9910P DFANHQMPSLSKLDKQMISYVEPGGNWTSIPESV--PSKRLDQIRAMAKTRGMVRTTYYS 714

M.Esp3I SFFNHGEPTLSELDRLIISYVPEGGNWQSIPETV--PSQRLKQIREMSAERGVVRTTYYG 726

M.BsmBI IVYNHTLPTLSELDKEMISYVKQGGNWEDIPETV--PSKRLEQIREMSKRRGKVRTTYYG 713

M.McaJXNU1ORF2865P VVYDHVSGKLSSLDEEVLRHVPPGGNWKDLPEDF--PSARVRQIREAFTRGEGSRSTYYG 62

IX

M.Bli388ORF2814P RLDPDQPSNTVTAYIF----NKFAHYS----EDRYITPREAARLQQFPDSHEFVGPITSV 111

M.HauORF2644P RLDPNKPSLTVTAFIF----NKFVHPF----ENRFITTREAARLQGFPDNFEFIGSLTSI 112

M.BssHII RLDPSKPSLTVTGYIF----NKFVHPY----ENRFITVREAARLQGFPDSLKFEGSLTST 125

M.Tbo1301ORF214495P RLDPNKPSLTVTGYIF----NKFVHPY----ENRFITVREAARLQGFPDGMKFEGTLTST 125

M.Afl2012ORF16135P RLDPDKPSLTVTGYIF----NKFVHPY----QDRFITVREAARLQGLPDDLQLRGTLTSS 125

M.Ski73ORF33040P RLDPEKPSLTVTGYIF----NKFVHPY----QNRFITVREAARLQGLPDDLKLQGTLTSC 125

M.EsaSS471P -----------------------------------LTPREAARIQSFPDDLIFEGNRREQ 25

M.Alw26I RINYEKPSYTITTYFNRPGNGTYVHPK----LERVITAREAARLQSFPDNYYFYGNKKDV 699

M.Ato52ORF13820P RINYEKPSYTITTYFNRPGNGTYVHPK----LERVITAREAARLQSFPDNYYFYGNKKDV 695

M.BsmAI RIDYDKPSYTITTYFNRPGNGTYVHPV----HDRVLSVREAARFQCFKDDYYFYGNKTQM 746

M1.Hpy655ORF350P RIDYNKPSYTITTCFNRPGNGTYVHPI----HNRVISVREAARFQTFQDDYYFYGNKKEI 110

M.Hpy173ORF5925P RIDCNKPSYTITTCFNRPGNGTYVHPI----HNRVISVREAARFQTFQDDYYFYGNKKEI 56

M1.Hpy328ORF5285P RIDYNKPSYTITTCFNRPGNGTYVHPI----HNRVISVREAARFQTFQDDYYFYGNKKEI 173

M2.BcoDI RLCWDKPSYTIATYFNRVGNGCNLHPS----QCRVLSNREAARLQSFPDSFIFQGSNASQ 110

M1.Bba133ORF575P RLEFDKPSYTITTYFNRLGNGCNLHPK----QDRIISTREGARLQSFRDSFIFLGSKTSQ 112

M2.Wsppc19ORF840P RLRYDKPSYTITTYFNRLGNSSNLHPE----QQRMISIREGARLQSFKDSFVFYGSKTSQ 110

M2.CstDORF413P RLTRDMPAYTISTNFNRPGCGCNTHYT----QDRTLTYREAARIQSFPDDFVFLGSKASV 114

M2.AfaJQ135ORF6300P RLHPNRPSYTINTYFTRPGNGCHIHYDYSGAQHRTLSHREAARLQSFPDNFLFKGNKGSV 136

M.HseOs45ORFCP RLHPDRPSYTINTYFTRPGNGCHIHYDYIGEQHRTLSHREAARLQTFPDNFVFKGSKGSI 678

M2.BsaI RLLPDMPSYTINTYFNRPGNGCHIHYE----QDRTLSQREAARLQSFPDDFIFYGSKTAI 100

M1.RxyORF2232P RLRPDAPAYTINTYFTRPGNGCHLHYDYSGGQHRTLSQREAARLQSFPDRFVFRGSHIAV 126

M2.Eco31I RLLPDMPAYTINTYFNRPGNGCHIHYE----QDRVLSQREAARLQSFPDDFIFFGGQTAI 121

M1.AadSW13ORF4345P RLKRDMPSYTINTYFNRPGNGCHIHFE----QNRVLSQREAARLQSFPDSHIFSGSMTAI 114

M.Ssp126ORF9910P RLKYTQPSYTISTYFNRPGNGANIHPW----EDRTLSCREAARLQSFPDSFKFLGNEAAV 770

M.Esp3I RLRRDQPAYTISTYFNRPGNGTHIHPV----LDRTLTSREAARIQSFPDRYIFLGSEGAV 782

M.BsmBI RLNPNQPAYTISTYFNRPGNGTNIHPW----ENRTISCREAARLQSFPDSFIFYGKEGAV 769

M.McaJXNU1ORF2865P RLTWQRPSYTISTYLTRPGNGSFIHPE----LPRLLTVREAARLQGFPDRIRFHGTLRQR 118

:: **.**:* : * : *

X

M.Bli388ORF2814P QLQIGNAVPVGLARAVARHVDARMKS-------------------N-NTCLDRKAVSLFS 151

M.HauORF2644P QRQIGNAVPVPLATAILQTILQHAKA-------------------HHPEKSLFSALSIFS 153

M.BssHII QMQVGNAVPVQLAKAVFEAVLISVRK-------------------LGYGKRNLTAFSLFS 166

M.Tbo1301ORF214495P QLQVGNAVPVPLAKAVFEHLAHQAHM-------------------LGFNNRTLKAFSLFS 166

M.Afl2012ORF16135P QLQVGNAVPVPLAKAVFQQLLKQANL-------------------LGFENRSLTALSLFS 166

M.Ski73ORF33040P QLQVGNAVPVPLAKAVFEQLVKQANL-------------------LGFENSSLTGLSLFS 166

M.EsaSS471P CILVGNAVPPLLAANLAKSILKHISESDGNIQNRPIQNKINFLNEKTSNMKSPTFVDLFS 85

M.Alw26I LTQIGNAVPCLFAQAIGSRLKEIVPT-------------------------LNTFGDLFA 734

M.Ato52ORF13820P LTQIGNAVPCLFAQAIGSRLKEIVPT-------------------------LNTFGDLFA 730

M.BsmAI LKQVGNAVPTILAYQIAKKIVDKTGC-------------------------R-KSIDLFC 780

M1.Hpy655ORF350P LNQVGNAVPVFLAYQIGKKIKDKIGC-------------------------Y-KSVDLFC 144

M.Hpy173ORF5925P LNQVGNAVPVFLAYQIGKKIKDKIGC-------------------------Y-KSVDLFC 90

M1.Hpy328ORF5285P LNQVGNAVPVFLAYQIGKKIKDKIGC-------------------------Y-KSVDLFC 207

M2.BcoDI YKQIGNAVPPLLARFVASLIMPHL-R-------------------------GMNFVDLFA 144

M1.Bba133ORF575P YKQIGNAVPPLLARAVAETLKPHL-Q-------------------------NQTFVDLFA 146

M2.Wsppc19ORF840P YKQIGNAVPTLLARAVAEVIYPHM-A-------------------------NKSMIDLFS 144

M2.CstDORF413P AKQIGNAVPPLLAYQVAKA----LPI-------------------------KGAFIDLFA 145

M2.AfaJQ135ORF6300P TTQIGNAVPPLLAFQVARH----LNI-------------------------VGQAVDLFA 167

M.HseOs45ORFCP ATQIGNAVPPLLAFQVAQH----LNI-------------------------VGQAVDLFA 709

M2.BsaI NNQIGNAVPPLLAYQIAKA----FPF-------------------------KGQFVDLFS 131

M1.RxyORF2232P SRQIGNAVPPLLAYQVARA----IPT-------------------------RGLFADLFS 157

M2.Eco31I NTQIGNAVPPFLAFLIAKEIEKAIGN-------------------------TGYYIDLFS 156

M1.AadSW13ORF4345P NNQIGNAVPPILAFQIAENLSKKLGK-------------------------KGYFIDLFC 149

M.Ssp126ORF9910P RTQIGNAVPPLLGYAIGKSIEEAVGR-------------------------TVKFCDVFA 805

M.Esp3I RDQIGNAVPPLLSSAIGRKLISY-AH-------------------------SKTCVDVFC 816

M.BsmBI RKQIGNAVPPLLSYALGKTI-----K-------------------------AKTFVDLFA 799

M.McaJXNU1ORF2865P CMQVGNAVPPVLAYQIGQVI-----E-------------------------SGTAVDLFA 148

:***** :. : .:*.

I

M.Bli388ORF2814P GAGGMDLGFSDYF----NIQS--ANEIESNFAATLARNFSS-----------THVLSSDV 194

M.HauORF2644P GAGGLNLGAQQAKLPNAKWQTIASIDIDRDACTSLEHHFAN----------KN-VICQNI 202

M.BssHII GAGGLDIGAEQATYKSMKIETLVTLDNWKDACDTLRGFYQG----------RTSVLQGDI 216

M.Tbo1301ORF214495P GAGGMDIGAHLTN----CIDICVTLDNWSDACATLGGFFRK----------GVSVLEKDI 212

M.Afl2012ORF16135P GAGGMDIGADLTQ----GIETKVAVDNWGDACATLKGYFGD----------RIQVIEQDI 212

M.Ski73ORF33040P GAGGMDIGADIA-----RIKTKVAVDNWGDACATLNGYFGD----------RIQVLEEDI 211

M.EsaSS471P GAGGITEGLLNA-----GYKGLLSVDFDQDCVNTHNLNHKN-----------IPGIKADL 129

M.Alw26I GAGGMSQGMFQA-----GLKPIFANDCFLSACISHKANHPE-----------TDVIYGDI 778

M.Ato52ORF13820P GAGGMSQGMFQA-----GLKPIFANDCFLSACISHKANHPE-----------TDVIYGDI 774

M.BsmAI GAGGLTAGFKEA-----GIQSVLCNDIEESACITLKINNPE-----------IKVLCGDI 824

M1.Hpy655ORF350P GAGGMTTGFKKA-----GIISLLGNDIDKSACITLKVNNPE-----------INVLCGDI 188

M.Hpy173ORF5925P GAGGMTTGFKKA-----GIISLLGNDIDKSACITLKVNNPE-----------INVLCGDI 134

M1.Hpy328ORF5285P GAGGMTTGFKKA-----GIISLLGNDIDKSACITLKVNNPE-----------INVLCGDI 251

M2.BcoDI GCGGMSEGFIMS-----GFQLIAANEIDKSIMATNRYNHSQYA-------PAENFILGDI 192

M1.Bba133ORF575P GAGGMSEGFISE-----GFKLIAANEIEKNYFETYKQNHLEFD-------NGDNLILGDI 194

M2.Wsppc19ORF840P GAGGMSEGFLME-----GFNVIGSNELEKHFFETYKQNHSSVN-------NEDSLILGDI 192

M2.CstDORF413P GAGGLALGFVWA-----GWQPVVSNDIVPDFLKTHAANIDG------------ATICGDI 188

M2.AfaJQ135ORF6300P GAGGLGLGFGWA-----GWTTLVGNELEASFAETYRTNVHS------------SIVVGDI 210

M.HseOs45ORFCP GAGGLGLGFGWA-----GWKTLVGNELEASFAETYRSNVHD------------DIVVGDI 752

M2.BsaI GAGGLSLGFLWA-----GWKPIIANDIDKWALTTYMNNIHN------------EVVLGDI 174

M1.RxyORF2232P GAGGLSLGFQWA-----GWQPVVANDVDEAALLTYRDNIHD------------VIVLGDI 200

M2.Eco31I GAGGLGLGFKWA-----GWTPLLANDIEEKYLQTYSNNVHK------------EVLCGSI 199

M1.AadSW13ORF4345P GAGGLGLGFKLN-----GWTSGLASDIDKNCVETYKNNLHE------------NVLLGDI 192

M.Ssp126ORF9910P GAGGLSYGMELA-----GFNGVAAIELNKDAAKTYSANHEN----------NITMVVGDI 850

M.Esp3I GAGGLSLGLESA-----GWNIVAAIDNNSDALDTYCFNRPCDLEPDNAQEGRTAVFKRDL 871

M.BsmBI GAGGLSYGFELA-----GLEGMAALEIDKDAAETYAKNHSS----------NIDVIVGDI 844

M.McaJXNU1ORF2865P GAGGLGLGLDWA-----GHEVIASVDNNRDACRTLAGCHGP----------SHRVLERDL 193

*.**: * : : . .:

IV

M.Bli388ORF2814P TDIDGS-------EL------SDSEVDLVFGGPPCQPFSAAGKHRGV--------DDPRG 233

M.HauORF2644P IDITQPKSLMS------------QPLDLSYGGPPCQSFSQAGKQKGL--------SDPRG 242

M.BssHII SEIQDPKLLWHQESQ------HDQIPDIVFGGPPCQAFSQAGKQKAT--------NDPRG 262

M.Tbo1301ORF214495P SALEDPLNLWQNASG------EVDKPDLVFGGPPCQAFSQAGKQKGL--------QDDRG 258

M.Afl2012ORF16135P SHITDPLSFWQNVSG------EINKPDIIFGGPPCQAFSQAGKQRGL--------QDKNG 258

M.Ski73ORF33040P SQITDPLSFWQNVSG------EINKPDIIFGGPPCQAFSQAGKQRGL--------EDQRG 257

M.EsaSS471P SQEKYEKLILEKI--------GDQKLDLLIGGPPCQGFSIFGKRRFVNSQKFQPNTDNRN 181

M.Alw26I SEAHTKQKIYQY--A--------NKIDILCGGPPCQGFSQAG-KRII--------DDPRN 819

M.Ato52ORF13820P SEAHTKQKIYQY--A--------NKIDILCGGPPCQGFSQAG-KRII--------DDPRN 815

M.BsmAI SQHETKEHIVNVAIN--------EDVDIICGGPPCQGFSMAG-LRLT--------DDPRN 867

M1.Hpy655ORF350P TQQAIKNKISSIALE--------QGADIICGGPPCQGFSMAG-FRAD--------NDPRN 231

M.Hpy173ORF5925P TQQAIKNKISSIALE--------QGADIICGGPPCQGFSMAG-FRAD--------NDPRN 177

M1.Hpy328ORF5285P TQQAIKNKISSIALE--------QGADIICGGPPCQGFSMAG-FRAD--------NDPRN 294

M2.BcoDI TQEETKARIMEAC-GN-------TPVNVVVGGPPCQGFSYAG-WRDP--------NDKRN 235

M1.Bba133ORF575P TLPEIKQQIVSVASKQ-------KKIGVVIGGPPCQGFSNAG-WRNP--------NDKRN 238

M2.Wsppc19ORF840P TDSIIKEKIINSKNKV-------DKVGLVIGGPPCQGFSYAG-WRNP--------NDTRN 236

M2.CstDORF413P SDPEILNSICDAAAKF--RKDNPDSPLFVLGGPPCQGFSTA-NYRSI--------QDARN 237

M2.AfaJQ135ORF6300P TDDSVKNQILKEVKGA----RNKDLPLCVLGGPPCQGFSTAGNKRSM--------DDERN 258

M.HseOs45ORFCP TESVTKEQILKQVRDA----RDSSLPLCVLGGPPCQGFSTAGNKRTM--------EDKRN 800

M2.BsaI RDEKVSETIIQKCLIA--KKSNPDRPLFVLGGPPCQGFSTAGKKRSI--------VDERN 224

M1.RxyORF2232P REHHVIDAIIQKCEEA--RDASPNMPFFVLGGPPCQGFSTAGNRRFV--------GDERN 250

M2.Eco31I SDNETFSKIADKISGF--KKLYFDKQLWILGGPPCQGFSTAGNARTM--------DDPRN 249

M1.AadSW13ORF4345P SDGEFFKKLVTASLLVKERAKKENAPFWILGGPPCQGFSTAGHKRTM--------DDERN 244

M.Ssp126ORF9910P NNESVQSEFISAIEKG----ISPDEPWVMVGGPPCQGFSTAG-YRDE--------NDIRN 897

M.Esp3I QERREFEDVVTRIETG----LGSTKLDLLVGGPPCQGFSHAG-YRLS--------DDKRN 918

M.BsmBI RSPEIQNQLIESVKNK----LKGRTLDLIAGGLPCQGFSTAG-WRKP--------DDERN 891

M.McaJXNU1ORF2865P SLPEHVEDVVTTVRSR----LAGRSLGLLAGGPPCQGFSTAG-PCRV--------DDPRN 240

** *** ** * ..

VI

M.Bli388ORF2814P GLVKEYFRIVSELSPSYFVLENVPGLLSNAKGGALKFVLEQAEKIGYA--SEYFVLNATD 291

M.HauORF2644P NLIYEFIRFLSDLNPNYFLLENVKGLQGINNGQLLHLIIDDIRKLGYN--VTFGVVNAAD 300

M.BssHII NLIYEYLRFIEKINPPFFVMENVANLKGVQRGELYQDILERMSNLGYN--VTVAPLLAAD 320

M.Tbo1301ORF214495P KMIYEFLRFVEHLHPPFFVMENVPNLKGIAGSQLYQEILQKMANLGYQ--VSVGILLAAD 316

M.Afl2012ORF16135P GMIYEFLRFIECLQPNFFVMENVSNLKGLGGGKLYQEIWENMINLGYN--VSSGVLLAAD 316

M.Ski73ORF33040P SMIYEFLRFVEYLQPAFFVMENVSNLKGLGGGKLYQEICNNMIDLGYN--VSSGVLLAAD 315

M.EsaSS471P DLVIKFWRYAELLKPNWVIMENVPGFASLLDGYYLEQSIKYAKKLGYKS-IDYKVINCAD 240

M.Alw26I QLFLEFIESISVINPKVVVMENVQGFLTLDKGNFYDQTKELLEELGYV--CEGRLLNTVH 877

M.Ato52ORF13820P QLFLEFIESISVINPKVVVMENVQGFLTLDKGNFYDQTKELLEELGYV--CEGRLLNTVH 873

M.BsmAI QLFKEFIEIVSRVKPKVIVFENVEGILSFQSGKVYRAILEMFSEIGYF--TEGRTLMSSD 925

M1.Hpy655ORF350P QLFRDFIDVIKKVKPKIIVFENVEGLLSYQKGKIYKEIHQLFSELGYN--TNGRVMSANE 289

M.Hpy173ORF5925P QLFRDFIDVIKKVKPKIIVFENVEGLLSYQKGKIYKEIHQLFSELGYN--TNGRVMSANE 235

M1.Hpy328ORF5285P QLFRDFIDVIKKVKPKIIVFENVEGLLSYQKGKIYKEIHQLFSELGYN--TNGRVMSANE 352

M2.BcoDI QLFKDFVEMVNRLRPEFFVMENVPGILTMRKGDAIKEIIEAFTEIGYRVNV-PIKLNAEE 294

M1.Bba133ORF575P QLFKEFVQIVDELQPEIFVMENVPGILTMRKGEAIKEIIASFEAIGYNINK-PFKLNAEE 297

M2.Wsppc19ORF840P QLFKDFVEVVRDVRPEGFVMENVPGILTMRKGEAVKEIISTFEGLGYNVNK-PFKLTAEE 295

M2.CstDORF413P WLFKEYTKVLAKVKPDGFIFENVAGILNFEKGTFFPRILSELGEHVEA--FKVNKVNCAN 295

M2.AfaJQ135ORF6300P WLFRDYCGLLAVLKPDVFVFENVTGLLSMEGGRVFEMVKDELSKHAKR--LIVWKLHSEN 316

M.HseOs45ORFCP WLFRDYCGLLDAIKPDVFVFENVTGLLSMEGGRVFEMVKEELSKHAKR--LIVWKLHSEN 858

M2.BsaI WLFESYVSILKEVKPDGFIFENVTGLLSMEKGAFFEMVKSELSKTVSN--LFVYKLNSVD 282

M1.RxyORF2232P WLFRQYKAVLERLQPDGFVFENVPGLLNMEGGSVFRAIRDELASTTKQ--LVVWRLRAEE 308

M2.Eco31I SLFMHYKSLLNEIKPNGFIFENVAGLLNMEKGKVFERVKEEFSSTMKT--MNGWILNSEH 307

M1.AadSW13ORF4345P HLFKHYNKLLGLVNPDGFVFENVAGLLSMQKGAVFEQVKKAFKENMKS--INAWLLHSEK 302

M.Ssp126ORF9910P KLVDSYLKLIQRVQPTIVVMENVQGILSMKGGKVIEGVYASLSKLGYKLNAEPWVLDAEM 957

M.Esp3I DLASIYLHFAERLRPRIFILENVEGLATFNKGQTLRDICTTLQELGYRVNIPVWKLCSEQ 978

M.BsmBI ALVTYFLQVVQKLMPNYVLIENVEGLINMNKGLVLKSIHEVLDELGYIYYKNPWVLSAEQ 951

M.McaJXNU1ORF2865P LLVLSFLEAVRQLRPERVLFENVLALRWRGKA-FLDELTERLASLGYD--VDIRVLHAEA 297

: : : * .::*** : . :

VIII

M.Bli388ORF2814P FGVPQLRNRLFVIGRRDKSEAPLGRPLATHCLPDR----------QG-NLLLPSCMTVGE 340

M.HauORF2644P YGTPQLRKRIIILGCKQDL-GFVNLPLPTHATEA------------N-LL-LQPYKTVGQ 345

M.BssHII YGAPQLRKRLIFLGCKKEF-GVMELPVPTHSNTP------------D-LLSPNPYVTVGE 366

M.Tbo1301ORF214495P FGTPQLRRRLFFVGCLKEI-GSISLPLPTHTPEP------------E-LFGLLPYVTVGQ 362

M.Afl2012ORF16135P FGTPQLRKRLFFIGCRKDI-GSIDLPLPTHGPKF------------E-LFGLLPYLTVQE 362

M.Ski73ORF33040P FGTPQLRKRLFFIGSRKHI-GSIDLPLPTHSPGC------------Q-LLGLLPYVTVGE 361

M.EsaSS471P YGVPQKRKRFIMFASKEKINLPWPKKKYFESPK---------------SWQTPYR-VVGD 284

M.Alw26I YGVPQKRKRVIILGVHKNLIGSHKIEEFFPTPT---------------TLDESQQVSAFE 922

M.Ato52ORF13820P YGVPQKRKRVIILGVHKNVIGSHKIEEFFPTPT---------------TLDESQQVSAFE 918

M.BsmAI YAVPQKRKRVFIICTRDDMD--VKPADLFPTPI---------------TEEPECQITARD 968

M1.Hpy655ORF350P FGVSQKRKRVIIICARDDLN--IIPSELFPQPI---------------TIEAKKQITAKD 332

M.Hpy173ORF5925P FGVSQKRKRVIIICARDDLN--IMPSELFPQPI---------------TIEAKKQITAKD 278

M1.Hpy328ORF5285P FGVSQKRRRVIIICARDDLN--IMPSELFPQPI---------------TIEAKKQITAKD 395

M2.BcoDI FGVPQRRKRVFIIGSLEEISIPQPSPLFYMPSV-----------KTPNMWNLPVAITVRD 343

M1.Bba133ORF575P FGVPQKRKRVVIIGSLKKEKINQPKKLFSSND-----------------ENLPKPINVKQ 340

M2.Wsppc19ORF840P YGVPQKRKRVFIVGTLSKIKINAPKALFSLKD-----------------ENLPNPITVKE 338

M2.CstDORF413P YGVPQRRERVIVLGAAQHVVDSFELAPITRIEV--RTKSISRITNAGVDESYPLVPSVMD 353

M2.AfaJQ135ORF6300P YVIPQRRTRVIIVGDNTGKVPESPPPMLSSLSA------------SDLVSDLPRPPSVKD 364

M.HseOs45ORFCP YAIPQRRSRVIIVGDNTGKIPDSPPKIISTLSA------------KDLISDLPQAPSVKD 906

M2.BsaI YGVPQRRNRVVIIGDSTGTKNSEPPIPITSLKG-----------EKTLFDALSSAISVKE 331

M1.RxyORF2232P YGVPQRRTRVILVGDSSGDVESEPPKTVTQLGN-----------NMSLFNELPAAVSVRD 357

M2.Eco31I YAIPQRRKRVILVGSNDPLFSI-EPPQ-----------------K--LTEDKESWVSVKD 347

M1.AadSW13ORF4345P YGVPQRRKRVFLVGFKEEEQVFYEPPQITSLPT-----------E--ASNNYSNSVSVGE 349

M.Ssp126ORF9910P YGVPQMRRRVIIVASKDEKYLPSVPEALFEKCLGRRETNDGQT----SLSFHRYPVTVGE 1013

M.Esp3I YGVPQMRRRIFVVATTDDTIDLSEPAPIYERCAGRRKNKIK---------TDLFSTNLPA 1029

M.BsmBI YGVPQMRKRVFIVAAKKGLELPKPPVQYFDKCLGRREKESDR-------KTDRYPVTVAE 1004

M.McaJXNU1ORF2865P YGVPQLRRRLVL------------------------------------------------ 309

: * * *...

M.Bli388ORF2814P AFAGLGKPTVRTLNRHKGVAVVDS------------------------------------ 364

M.HauORF2644P ALQNLPPALVWQKTTTK------------------------------------------- 362

M.BssHII AFKGLPKLV--------------------------------------------------- 375

M.Tbo1301ORF214495P AFADLPEAEFSR------------------------------------------------ 374

M.Afl2012ORF16135P AFANLPEAEFSRCR---------------------------------------------- 376

M.Ski73ORF33040P AFANLPDAEFSRCR---------------------------------------------- 375

M.EsaSS471P VITDLSNESS----YDSKINHIPSNH-SDLVKER---YSYIERR---------------- 320

M.Alw26I AIADLEHVIPNEFIEKP--S-----TTNRYLDQINKY----------------------- 952

M.Ato52ORF13820P AIADLEHVIPNEFIEKP--S-----TTNRYLDQINKY----------------------- 948

M.BsmAI TIKDLENIQCDEKACYV--KVEHESDILKVFKGKMTYQEIY------------------- 1007

M1.Hpy655ORF350P TIKDLEIIECSESAKYK--SNNVNTATIDFLRNHLSYEDYIAKIQD-------------- 376

M.Hpy173ORF5925P TIKDLEIIECSESAKYK--SNNVNTATIDFLRNHLSYEDYIAKIQD-------------- 322

M1.Hpy328ORF5285P TIKDLEIIECSESAKYK--SNNVNTATIDFLRNHLSYEDYIAKIQD-------------- 439

M2.BcoDI AIGSLPELENGGGSLEMDYEPVQASAYDRLMYGELTFEEFYNLL---------------- 387

M1.Bba133ORF575P AIGSLPELKTGEGEFEMVCNYKPISAYEKLMSGEIDFTEFYERCSEKLPVAFSNT----- 395

M2.Wsppc19ORF840P AIGGLPKIETDSGAFEVESEYKSTSPYESMMMGEIDFKEFYELMKSKINNLSYVV----- 393

M2.CstDORF413P AIGDLPEVAAGEDASAEEYACAPSCEYQKLMRGLTDPASYVERYQNMSS----------- 402

M2.AfaJQ135ORF6300P ALDDLPALLPGQDGGDLGYRHRAMTPYQAFMRGEITAAQYLAEVTR-------------- 410

M.HseOs45ORFCP ALDDLPALLPGQDGSDLGYRHEATTPFQALMRGEITAAQYLGRVTR-------------- 952

M2.BsaI ALSDLPLLSPNEDGSWKNYVCEPQNIYQSFMRKKITAQQYIEMLSSLAII---------- 381

M1.RxyORF2232P ALSDLPALRPGEDGSHKDYTHDPEHPYQEFVRGLIGPEEYLRALNDRL------------ 405

M2.Eco31I ALSDLPPLQHGEDGSGKYYIHHPENDYQLFMRGNITPSEYYERNIKPSL----------- 396

M1.AadSW13ORF4345P AISDLPPLKHNEDASHKEYLHPPINAYQKFMRGHISAINYLSEMQG-------------- 395

M.Ssp126ORF9910P AFWGLSALMPVNTYFPK--DAVIDPTYSKWCSGEISTEEFLEIR---------------- 1055

M.Esp3I PFTVLDALSGLS--F----PENKSGKLQEWLVNLANIT---------------------- 1061

M.BsmBI AFFGLPCLLSPV--FTP--PLEINPLYSQWCNNIITTEEFLNKRGKIKIEQEELDAPQLK 1060

M.McaJXNU1ORF2865P ------------------------------------------------------------ 309
