# Supplementary figures and images for: Circularly permuted variants of two CG-specific prokaryotic DNA methyltransferases
Source: PLoS One. 2018 May 10;13(5):e0197232. doi: 10.1371/journal.pone.0197232 (PMC5944983; doi:10.1371/journal.pone.0197232)

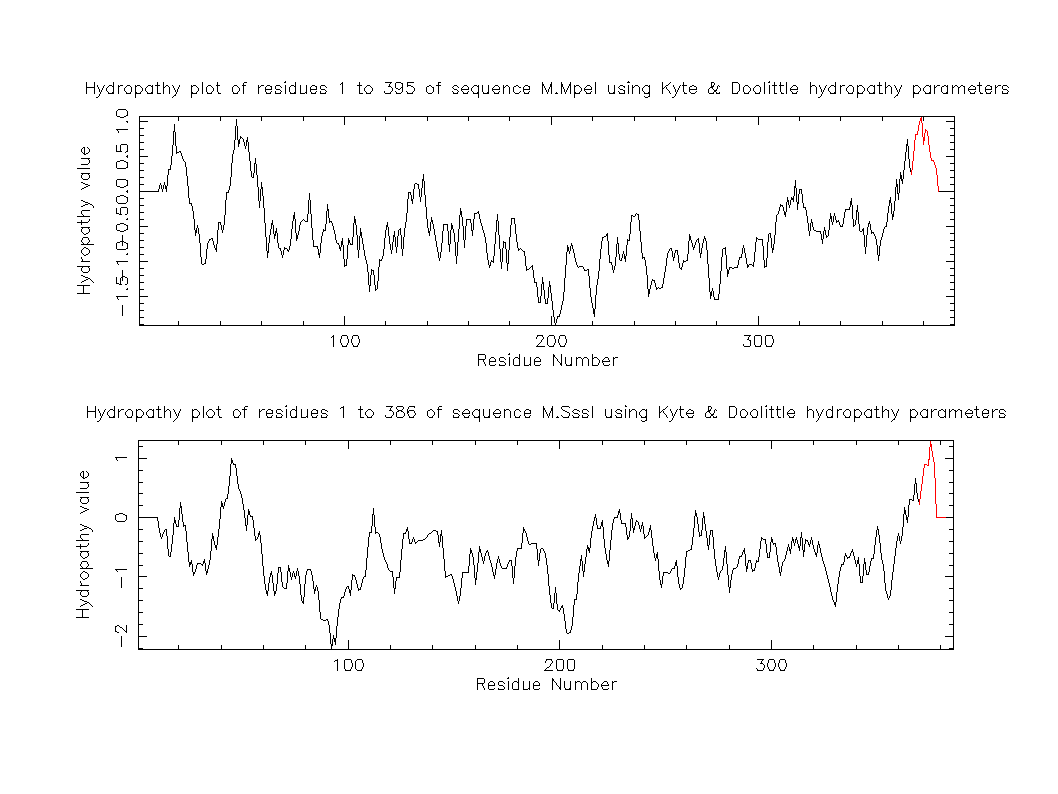

Supplement: S1 Fig — The hydropathy values of the residues located in the C-terminal α-helix are plotted in red. The plots were generated by the EMBOSS Pepinfo service (https://www.ebi.ac.uk/Tools/seqstats/emboss_pepinfo/) using Kyte and Doolittle parameters [42]. (TIF) [file pone.0197232.s007.tif]

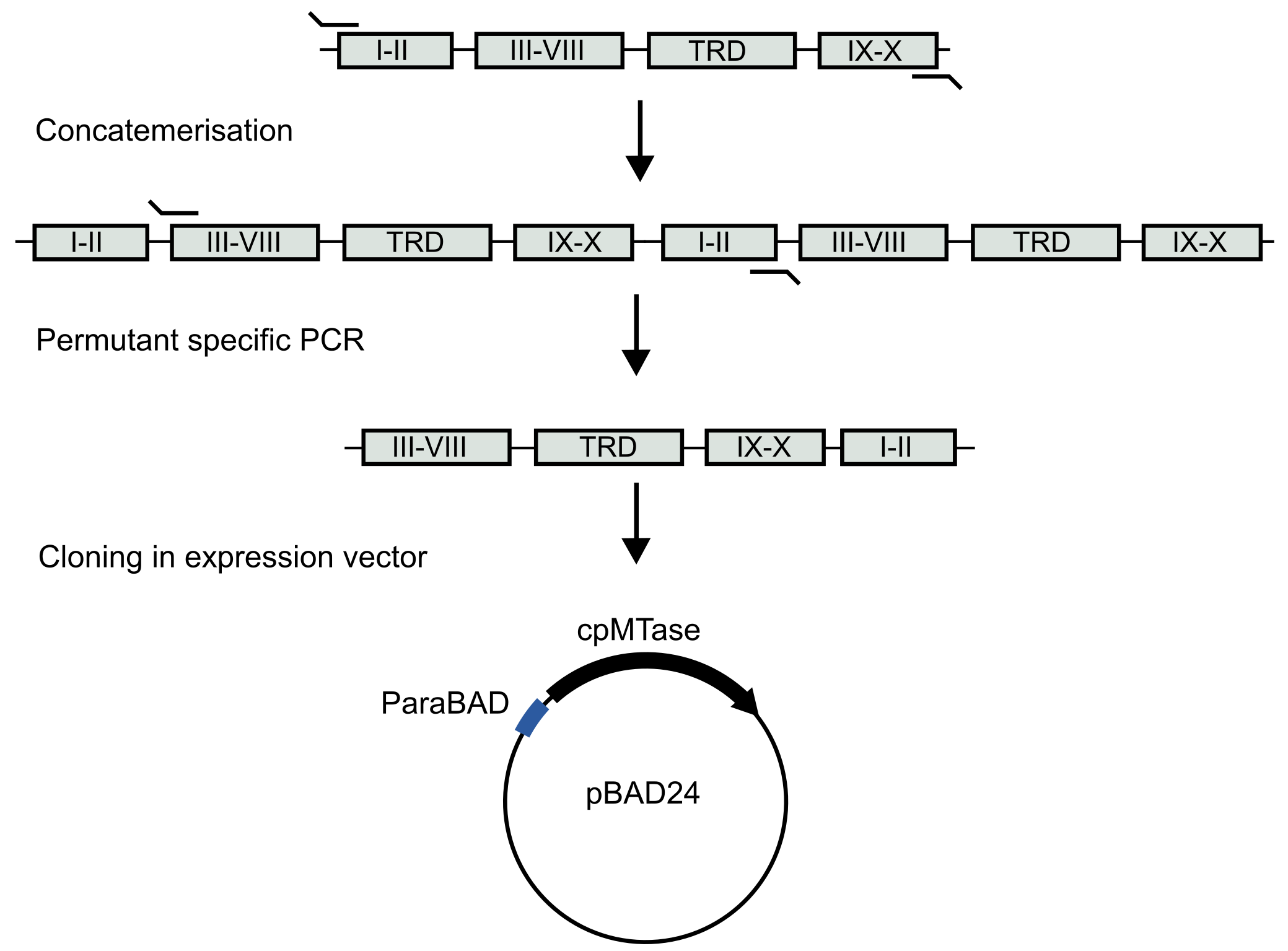

Supplement: S2 Fig — (TIFF) [file pone.0197232.s008.tiff]

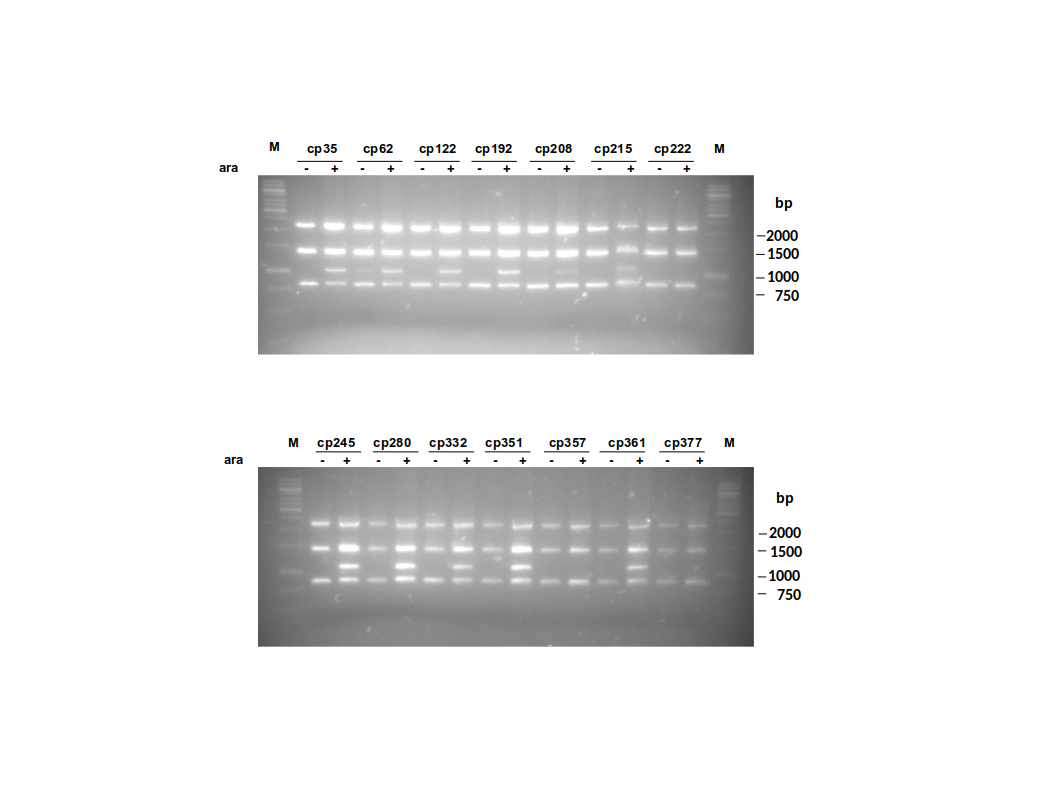

Supplement: S3 Fig — The plasmids contain five Eco47I sites. Uninduced and arabinose-induced cultures are indicated by minus and plus signs, respectively. Appearance of a 1059 bp fragment indicates methylation of a CG site overlapping one of the Eco47I sites in the plasmid. M, GeneRuler 1 kb DNA Ladder. (TIF) [file pone.0197232.s009.tif]

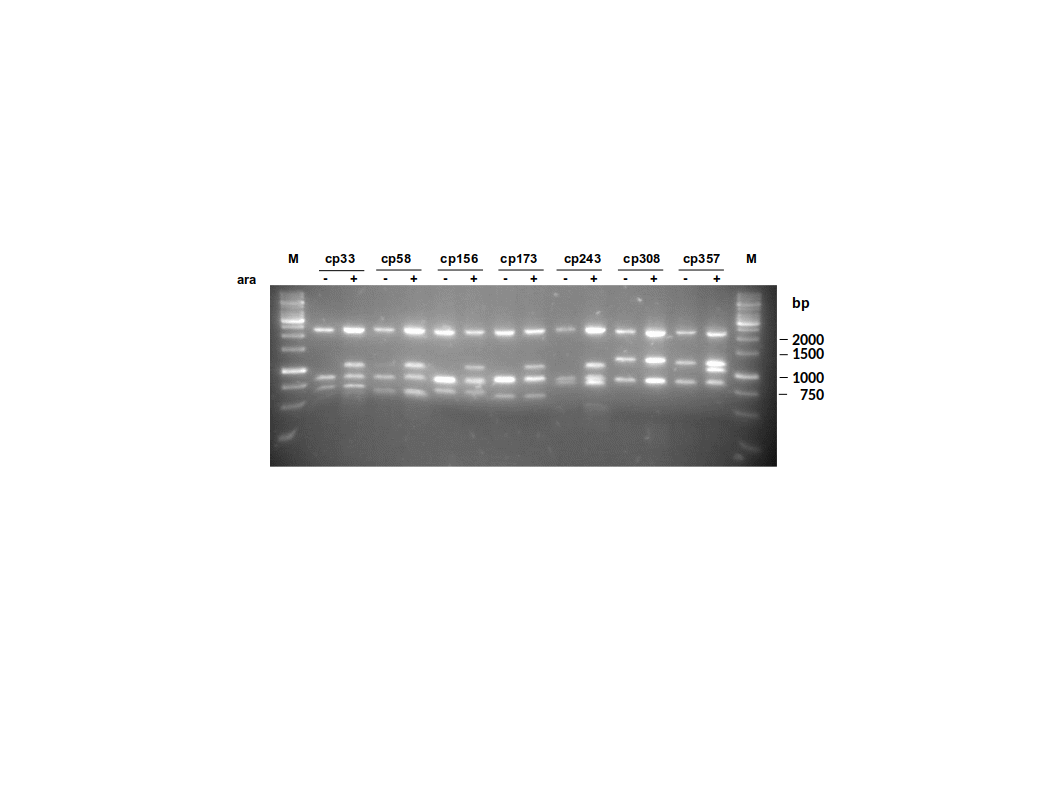

Supplement: S4 Fig — The plasmids contain six Eco47I sites. Uninduced and arabinose-induced cultures are indicated by minus and plus signs, respectively. Appearance of a 1059 bp fragment indicates methylation of a CG site overlapping one of the Eco47I sites in the plasmid. M, GeneRuler 1 kb DNA Ladder. (TIF) [file pone.0197232.s010.tif]

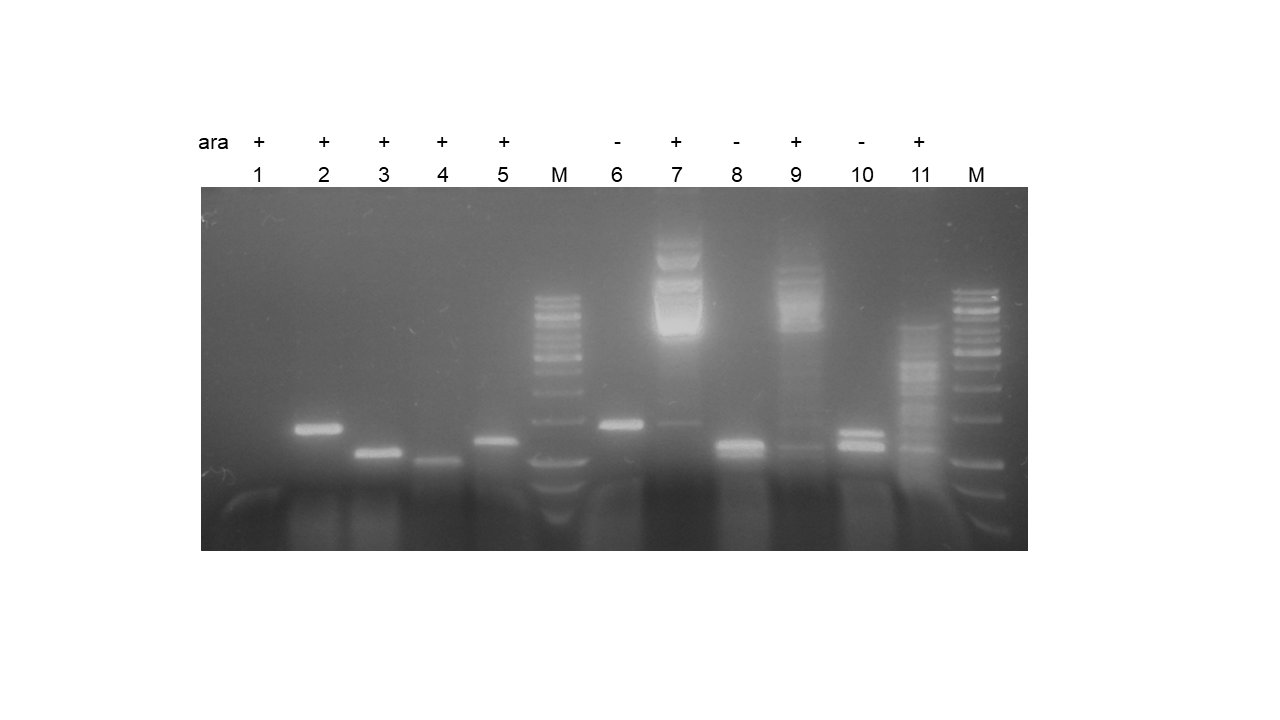

Supplement: S5 Fig — Plasmids were digested with Hin6I. Lane 1, pB-Mpe[1–61] Lane 2, pOB-Mpe[62–395] Lane 2, pOB-Mpe[62–395] Lane 3, pOB-Mpe[62–279] Lane 3, pOB-Mpe[62–279] Lane 4, pB-Mpe[280–61] Lane 5, pB-Mpe[192–61] Lanes 6 and 7, pB-Mpe[1–61] + pOB-Mpe[62–395] Lanes 8 and 9, pB-Mpe[280–61] + pOB-Mpe[62–279] Lanes 10 and 11, pB-Mpe[192–61] pOB-Mpe[62–279] M, 1 kb GeneRuler Induced cultures were grown in the presence of 0.1% arabinose at 30°C for 5 hours. (TIF) [file pone.0197232.s011.tif]

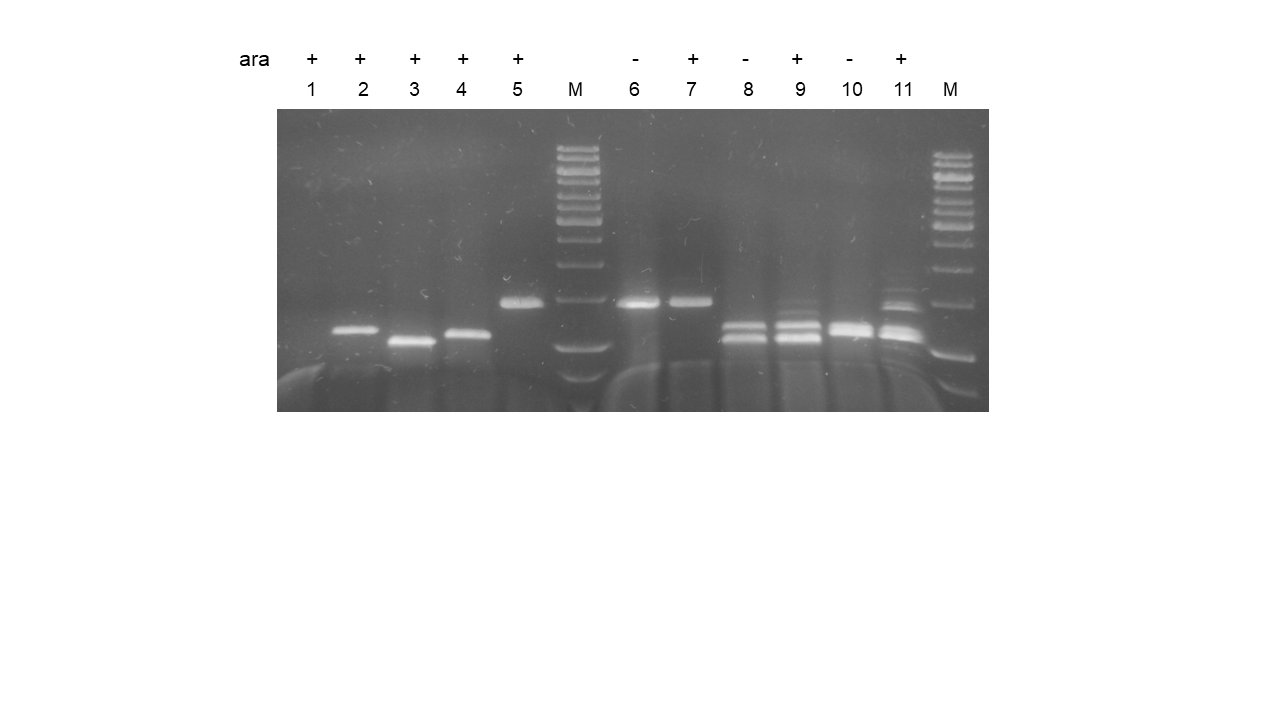

Supplement: S6 Fig — Plasmids were digested with Hin6I. Lane 1, pB-Sss[1–57] Lane 2, pB-Sss[58–275] Lane 3, pOB-Sss[276–57] Lane 4, pOB-Sss[243–57] Lane 5, pOB-Sss[58–386] Lanes 6 and 7, pB-Sss[1–57] + pOB-Sss[58–386] Lanes 8 and 9, pB-Sss[58–275] + pOB-Sss[276–57] Lanes 10 and 11, pB-Sss[58–275] + pOB-Sss[243–57] M, 1 kb GeneRuler Induced cultures were grown in the presence of 0.1% arabinose at 30°C for 5 hours. (TIF) [file pone.0197232.s012.tif]

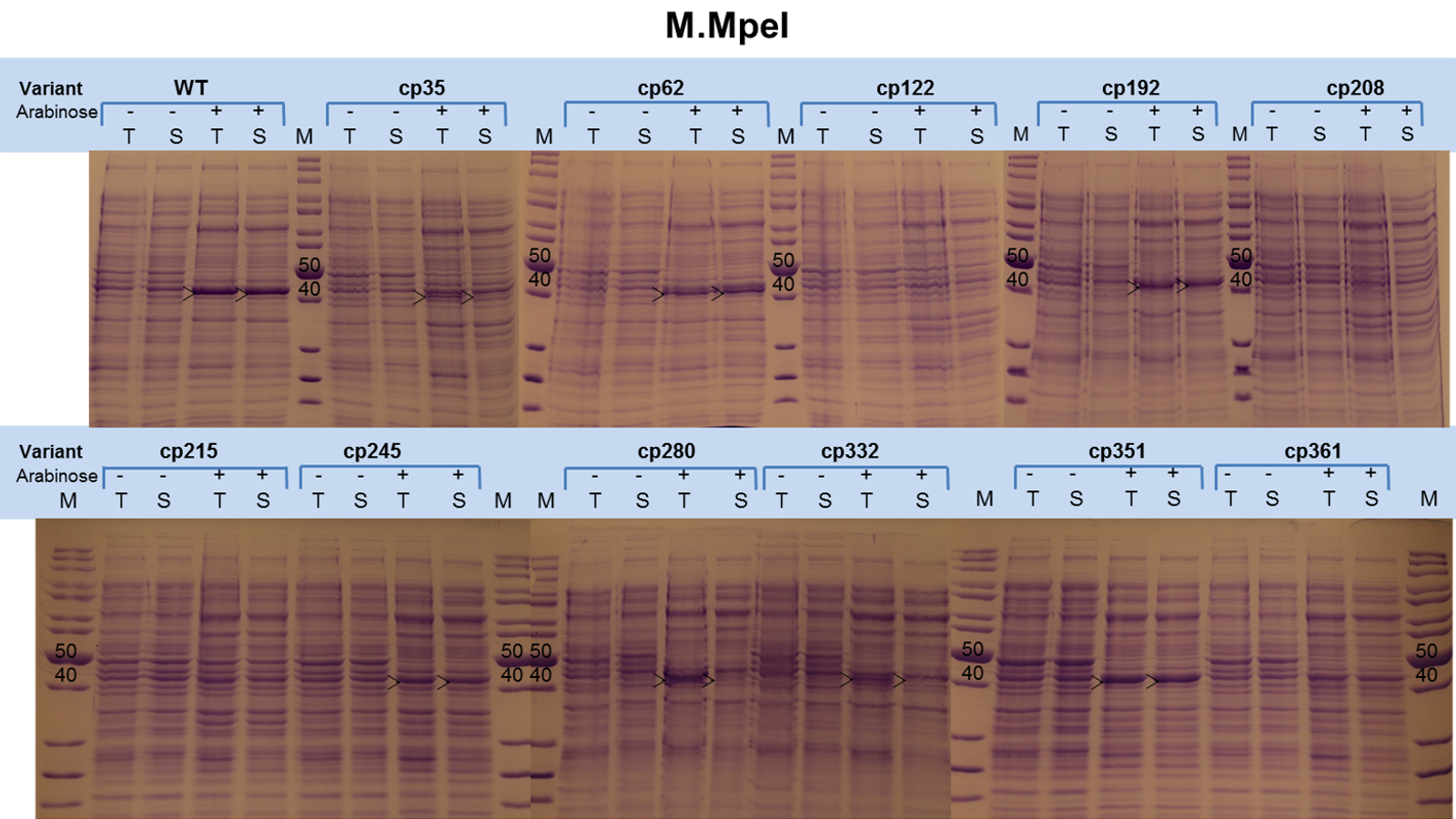

Supplement: S7 Fig — S, soluble fraction; T, total extract. M, molecular weight marker. Bands corresponding to the cpM.MpeI variants are indicated by arrowhead. (TIFF) [file pone.0197232.s013.tiff]

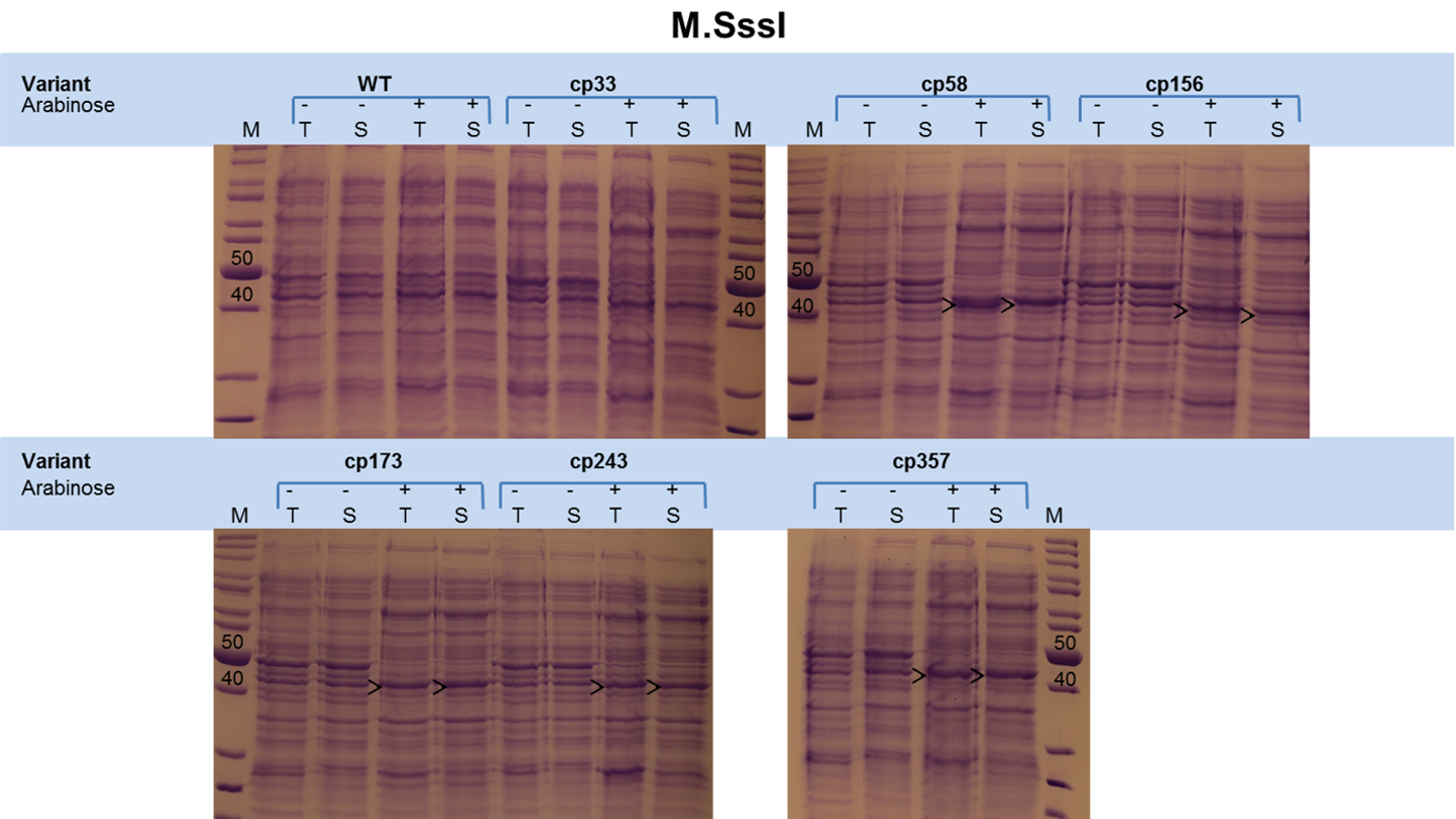

Supplement: S8 Fig — S, soluble fraction; T, total extract. M, molecular weight marker. Bands corresponding to the cpM.SssI variants are indicated by arrowhead. (TIFF) [file pone.0197232.s014.tiff]

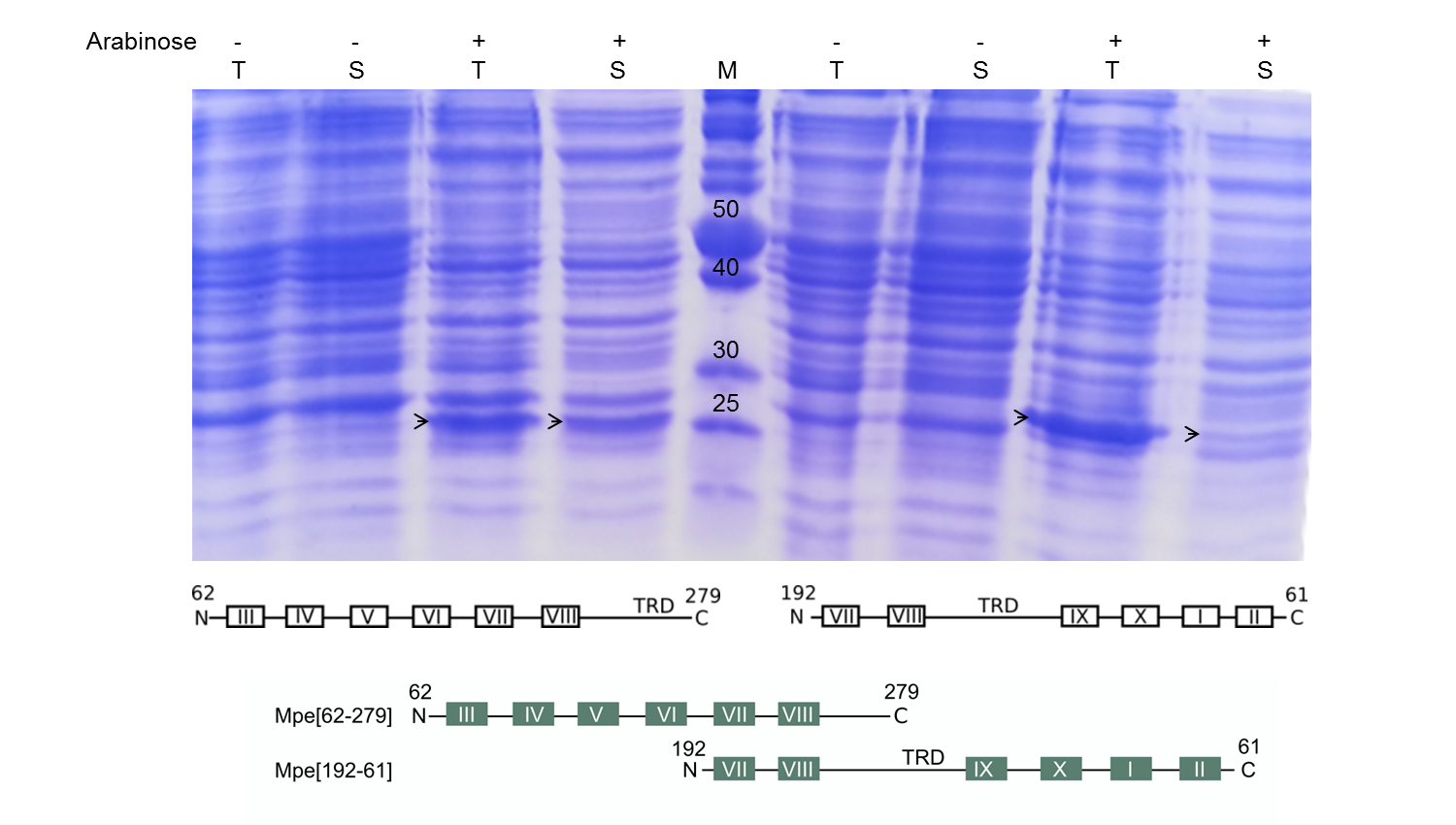

Supplement: S9 Fig — S, soluble fraction; T, total extract. M, molecular weight marker. Bands correspondig to the overproduced fragments are marked by arrowhead. The scheme under the gel shows the arrangement of the conserved motifs in the fragments. The fragments are inactive by themselves (empty boxes), but can assemble to produce a low activity enzyme when produced in the same E. coli cell (filled boxes). (TIFF) [file pone.0197232.s015.tiff]

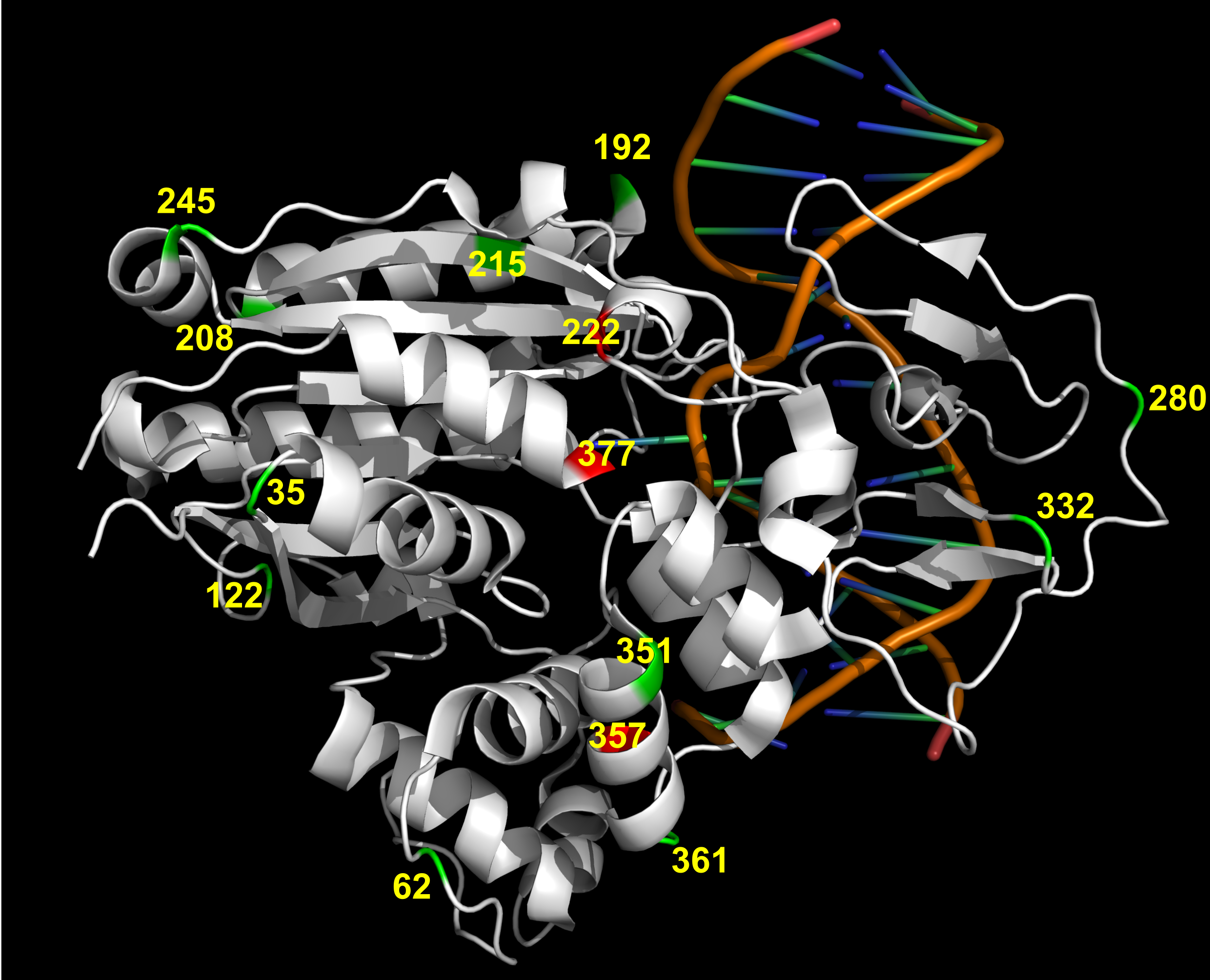

Supplement: S10 Fig — The sites yielding active and inactive MTase are highlighted in green and red, respectively. The yellow numbers indicate the positions of the N-terminal amino acids of the CP variants. (TIFF) [file pone.0197232.s016.tiff]

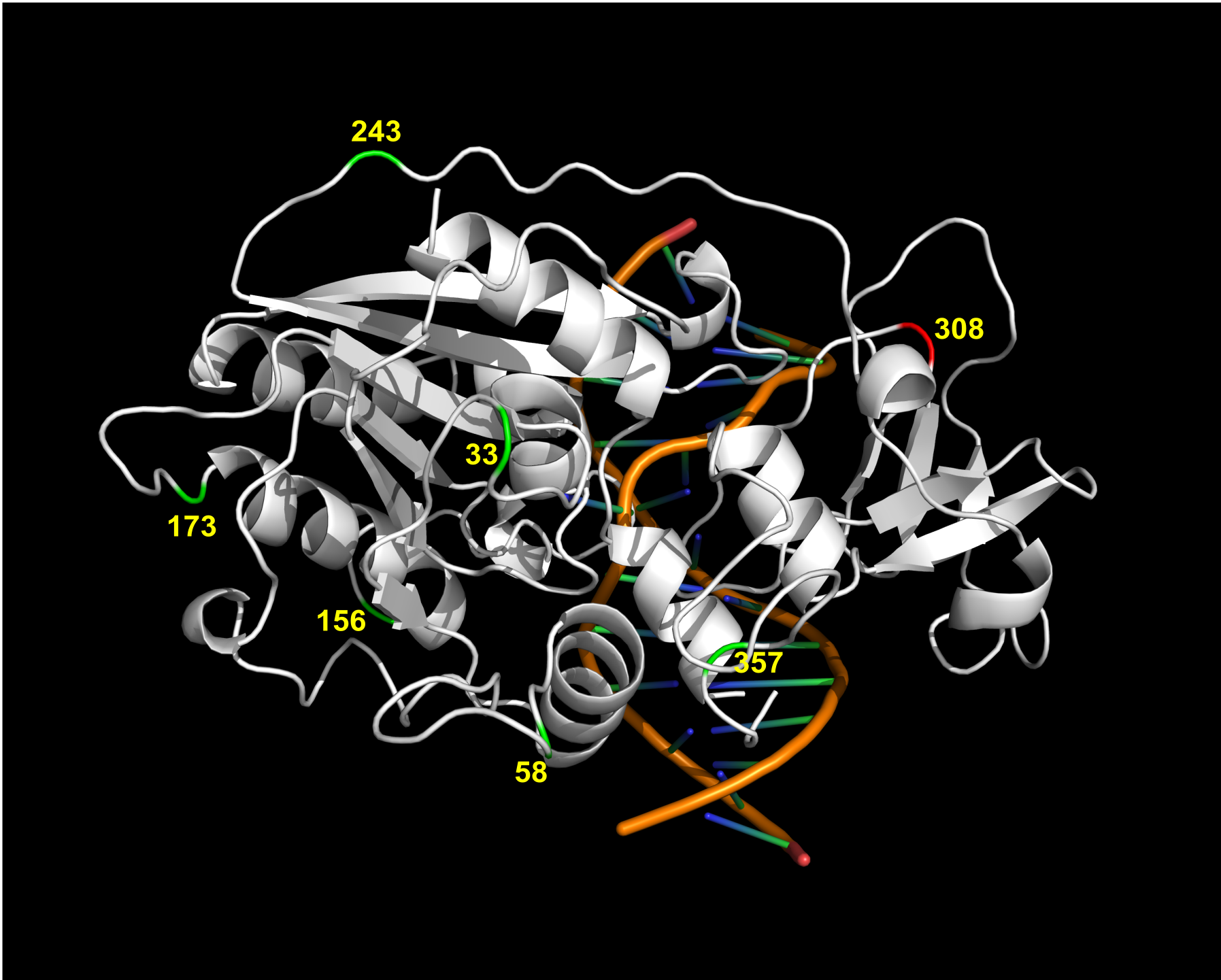

Supplement: S11 Fig — The sites yielding active and inactive MTase are highlighted in green and red, respectively. The yellow numbers indicate the positions of the N-terminal amino acids of the CP variants. (TIFF) [file pone.0197232.s017.tiff]
