# Supplementary material for: Circularly permuted variants of two CG-specific prokaryotic DNA methyltransferases
Source: PLoS One. 2018 May 10;13(5):e0197232. doi: 10.1371/journal.pone.0197232 (PMC5944983; doi:10.1371/journal.pone.0197232)
Supplement: S14 Fig — Clustal-Omega alignment. (DOCX) [file pone.0197232.s020.docx]

M.MpeI **35**

I II

M.MpeI -MNSNKDKIKVIKVFEAFAGIGSQFKALKNIARSK**N**WEIQHSGMVEWFVDAIVSYVAIHS 59

M.SssI -MSKVENKTKKLRVFEAFAGIGAQRKALEKVRK---**D**EYEIVGLAEWYVPAIVMYQAIHN 56

M.HhaI MIEIKDKQLTGLRFIDLFAGLGGFRLALESCGA------ECVYSNEWDKYAQEVYEM--- 51

M.HaeIII -----------MNLISLFSGAGGLDLGFQKAGF------RIICANEYDKSIWKTYES--- 40

:..:. *:* *. .::. . *: *

M.SssI **33**

M.MpeI **62**

M.MpeI KN**F**NPKIEQ-----------LDKDILSISNDSKMPISE-YGIKKINNTIKASYLNY-AKK 106

M.SssI N**F**-HTKLEYKSVSREEMIDYLENKTL--SWNSKNPVSNGYWKRKKDDELKIIYNAIKLSE 113

M.HhaI ------------------------------------------------------NFGE-K 56

M.HaeIII ------------------------------------------------------NHS**A**K**L** 46

M.SssI **58**

M.HaeIII  **44 46**

M.MpeI **122**

III IV V

M.MpeI HFNNLFDIKKVNKDN**F**PKNIDIFTYSFPCQDLSVQGLQKGIDKELNTRSGLLWEIERILE 166

M.SssI KEGNIFDIRDLYKR-TLKNIDLLTYSFPCQDLSQQGIQKGMKR**G**SGTRSGLLWEIERALD 172

M.HhaI PE---GDITQVNEKTIP-DHDILCAGFPCQAFSISGKQKGFEDS---RGTLFFDIARIVR 109

M.HaeIII IK---GD**I**SK**I**SSD**E**FP-KCDGIIGGPPCQSWSEGGSLRGIDDP---RGKLFYEYIRILK 99

** .: . . * : . *** * * :*:. *. *::: * :

M.SssI **156**

M.HaeIII **51 54 58**

M.MpeI 192 **208** **215** **222**

VI VII VIII

M.MpeI EIKNSFSKEEMPKYLLMENVKNLLS**H**KNKKNYNTWLKQLEK**F**GYKSKT**Y**LLNSKN**F**DNCQ 226

M.SssI **S**----TEKNDLPKYLLMENVGALLHKKNEEELNQWKQKLESLGYQNSIEVLNAADFGSSQ 228

M.HhaI E--------KKPKVVFMENVKNFASHDNGNTLEVVKNTMNELDYSFHAKVLNALDYGIPQ 161

M.HaeIII Q--------KKPIFFLAENVKGMMAQRHNKAVQEFIQEFDNAGYDVHIILLNANDYGVAQ 151

. . * .: *** : : : : : : ::. .*. :**: ::. *

M.SssI **173**

M.MpeI **245**

M.MpeI NRERVFCLSIRDDYLEKTGFKFKELEKVKNPPKKIKDILVDS--SNYKYLNLNK------ 278

M.SssI ARRRVFMISTLNEF**V**EL--------PKGDKKPKSIKKVLNKIVSEKDILNNLLK------ 274

M.HhaI KRERIYMICFRNDLNIQ-NFQ---FPKPFELNTFVKDLLLPDSEVEHLVIDR-------- 209

M.HaeIII DRKRVFYIGFRKELNIN-YLP----PIPHLI**K**P**T**FKDVIWDLKDN**P**IPALDK**N**KTNGNKC 206

*.*:: : .: .*.:: :

M.SssI **243**

M.HaeIII 178 180 **192**  **199**

M.Mpe **280**

TRD

M.MpeI -Y**E**TTTF-RETKSNII-SRS-----------LKNYTTFNSENYVYNINGIGPTLTASGAN 324

M.SssI -YNLTEF-KKTKSNIN-KAS-----------LIGYSKFNSEGYVYDPEFTGPTLTASGAN 320

M.HhaI ------------KDLVMTNQEIEQTTPKTVRLGIVGKGGQGERIYSTRGIAITLSAYGGG 257

M.HaeIII I**Y**PNHEYFIGSY***STIFMSRNR***VRQWNEPAFTVQA---***SGRQ*CQ**LHPQA**P**V**M**LKV------ 257

. : . . : . :: .:

M.SssI **308**

M.HaeIII **208** **245** 252

**246** 254

M.Mpe  **332**  **351**  **357** 361 377

IX X

M.MpeI ------SRIKIET**Q**QGVRYLTPLECFKYMQFD**V**NDFKK**V**QST**N**LISENKMIYIAGNSI**P**V 378

M.SssI ------SRIKIKDGSNIRKMNSDETFLYMGFDSQDGKRVNEI**E**FLTENQKIFVCGNSISV 374

M.HhaI IFAKTGGYLVNG---KTRKLHPRECARVMGYPDSYKV-----HPS-TSQAYKQFGNSVVI 308

M.HaeIII --SKNL**N**KFVEGKEHLYRRLTVRECARVQGFPDDFIF-----HYESLNDGYKMIGNAVPV 310

. : * : * : . . .. **:: :

M.SssI **357**

M.HaeIII **262**

M.MpeI KILEAIFNTLEFVNNEE--- 395

M.SssI EVLEAIIDKIGG-------- 386

M.HhaI NVLQYIAYNIGSSLNFKPY- 327

M.HaeIII NLAYEIAKTIKSALEICKGN 330

:: * .:

**S14 Figure.** Permutation sites of circularly permuted variants of M.MpeI, M.SssI and M.HaeIII. Clustal-Omega alignment.

Yellow highlighting: conserved motifs (Koudan et al.)

Red highlighting: N-termini of cpM.MpeI variants (this work)

Turquoise highlighting: N-termini of cpM.SssI variants (this work)

Purple highlighting: N-termini of cpM.HaeIII variants (Peisajovich et al.)

***S_219_TIFMSRNR_227_……S_241_GRQ_244_*** amino acids important for DNA recognition by M.HaeIII (Reinisch et al.)

**References**

Koudan EV, Bujnicki JM, Gromova ES. Homology modeling of the CG-specific DNA methyltransferase SssI and its complexes with DNA and AdoHcy. J Biomol Struct Dyn. 2004;22(3):339-45.

Peisajovich SG, Rockah L, Tawfik DS. Evolution of new protein topologies through multistep gene rearrangements. Nat Genet. 2006;38(2):168-74.

Reinisch KM, Chen L, Verdine GL, Lipscomb WN. The crystal structure of Haelll methyltransferase covalently complexed to DNA: An extrahelical cytosine and rearranged base pairing. Cell. 1995;82(1):143-53.
