# Supplementary material for: Circularly permuted variants of two CG-specific prokaryotic DNA methyltransferases
Source: PLoS One. 2018 May 10;13(5):e0197232. doi: 10.1371/journal.pone.0197232 (PMC5944983; doi:10.1371/journal.pone.0197232)
Supplement: S1 Table — (DOCX) [file pone.0197232.s021.docx]

**S1 Table**. **Oligonucleotides used in this work.**

| **Name** | **Sequence** | **Use** |
| --- | --- | --- |
| AK92 | gattagcggatcctacctga | Sequencing primers for pBAD24 and pOK-BAD. |
| AK147 | AGACCGCTTCTGCGTTCTGA |  |
| AK280 | TCGAAGGCGGTGGCAGCGGTC | Linker encoding GGGSG |
| AK281 | TCGAAGGCGGTGGCAGCGGTC |  |
| AK387 | ATCTCGAG**ATG**GGCAATAGCAAC | Primers for amplification of the M.MpeI gene |
| AK388 | ATGCTGAGCCTCGAGTTCCTCATTAT |  |
| AK391 | ATCC**ATG**GTAAAAATCTTAGAAGCTAT | Primers for amplification of cp377M.MpeI |
| AK392 | TATAAGC**TTA**AGGTATTGAGTTTCCTG |  |
| AK393 | ATCC**ATG**GGTTTTAAATTTAAAGAA | Primers for amplification of cp245M.MpeI |
| AK394 | TATAAGC**TTA**TGTTTTTTCTAGATAATCA |  |
| AK395 | ATCC**ATG**GAAACCACTACTTTTAGA | Primers for amplification of cp280M.MpeI |
| AK396 | TCTAAGC**TTA**ATATTTGTTTAAATTTAGATAT |  |
| AK397 | ATCC**ATG**GTTCAATCAACTAATTTAA | Primers for amplification of cp357M.MpeI |
| AK398 | TCTAAGC**TTA**TTTTTTAAAATCATTAACAT |  |
| AK413 | ATCC**ATG**GATGAGCAAAGTAGAAAA | Primers for amplification of the M.SssI gene |
| AK414 | ATCTCGAGACCTCCAATTTTATCTAT |  |
| AK415 | ATCC**ATG**GGTAATTGGGAGATTCAAC | Primers for amplification of cp35M.MpeI |
| AK416 | TCCAAGC**TTA**TTTGCTTCTTGCAATA |  |
| AK417 | ATCC**ATG**GGTTTTAATCCTAAAATTGAG | Primers for amplification of cp62M.MpeI |
| AK418 | TCTAAGC**TTA**ATTTTTTGAATGAATTG |  |
| AK419 | ATCC**ATG**GGTTTTCCTAAAAACATTG | Primers for amplification of cp122M.MpeI |
| AK420 | TCCAAGC**TTA**ATTATCTTTGTTAACTTTTT |  |
| AK421 | ATCC**ATG**GGTCAACAAGGTGTAAGATAT | Primers for amplification of cp332M.MpeI |
| AK422 | TCCAAGC**TTA**AGTTTCAATTTTAATTCT |  |
| AK423 | ATCC**ATG**GTTAATGATTTTAAAAAAG | Primers for amplification of cp351M.MpeI |
| AK424 | TATAAGC**TTA**ATCAAATTGCATATACTT |  |
| AK425 | ATCC**ATG**GGTAATTTAATTTCTGAAAAT | Primers for amplification of cp361M.MpeI |
| AK426 | TATAAGC**TTA**AGTTGATTGAACTTTTTT |  |
| AK428 | ATCC**ATG**GTTGAACTACCAAAGG | Primers for amplification of cp243M.SssI |
| AK429 | TATGTCGA**CTA**AAATTCATTTAAAGTAGAT |  |
| AK431 | ATCC**ATG**GATGAATATGAAATAGTAGG | Primers for amplification of cp33M.SssI |
| AK432 | TATGTCGA**CTA**TTTTCTGACTTTCTCCA |  |
| AK433 | ATCC**ATG**GGTTCTGGTACTAGATCAGGT | Primers for amplification of cp156M.SssI |
| AK434 | TATGTCGA**CTA**ACCTCTTTTCATACCCTT |  |
| AK435 | ATCC**ATG**GGATCAACTGAAAAAAATGA | Primers for amplification of cp173M.SssI |
| AK436 | TATGTCGA**CTA**ATCCAAAGCTCTTTCG |  |
| AK437 | ATCC**ATG**GAATTTACAGGACCAAC | Primers for amplification of cp308M.SssI |
| AK438 | TATGTCGA**CTA**AGGATCATAAACATAACC |  |
| AK442 | ATCC**ATG**GGTCATAAAAACAAAAAAAAC | Primers for amplification of cp192M.MpeI |
| AK443 | GCCAAGC**TTA**ACTTAATAAGTTTTTAACAT |  |
| AK444 | ATCC**ATG**GGTTTTGGTTACAAATCTAAA | Primers for amplification of cp208M.MpeI |
| AK445 | TCCAAGC**TTA**TTTTTCTAACTGCTTTAA |  |
| AK446 | ATCC**ATG**GAATTTTTAACTGAAAATC | Primers for amplification of cp357M.SssI |
| AK447 | TATGTCGA**CTA**AATTTCATTTACTCTTTT |  |
| AK448 | ATCC**ATG**GGATTTCATACAAAGTTGG | Primers for amplification of cp58M.SssI |
| AK449 | TATGTCGA**CTA**ATTGTTGTGTATAGCTTG |  |
| AK452 | ATCC**ATG**GGTTTTGATAATTGTCAAAA | Primers for amplification of cp222M.MpeI |
| AK453 | TCCAAGC**TTA**ATTTTTAGAATTTAATAAATATG |  |
| AK461 | ATCC**ATG**GGTTATTTATTAAATTCTAAAAAT | Primers for amplification of cp215M.MpeI |
| AK462 | TCCAAGC**TTA**TGTTTTAGATTTGTAACC |  |
| AK463 | CCATACC**ATG**GGCAATAG | Forward primer for amplification of Mpe[1-61] |
| AK464 | TCTAAGC**TTA**CTCGAGTTCCTCATTATT | Reverse primer for amplification of Mpe[62-395] |
| AK467 | GCTGTCGA**CTA**ATATTTCAATAAATTATTTAAA | Reverse primer for amplification of Sss[58-275] |
| AK468 | ATCC**ATG**GGAAATTTAACTGAATTTAAAAA | Forward primer for amplification of Sss[276-57] |

Restriction sites incorporated into the primers to facilitate cloning of the PCR products are underlined: CCATGG, NcoI; AAGCTT, HindIII; CTCGAG, XhoI; GTCGAC, SalI. The ATG start codons in forward primers and the complement of the stop codons in reverse primers are shown in green and red, respectively.
