# Supplementary material for: Circularly permuted variants of two CG-specific prokaryotic DNA methyltransferases
Source: PLoS One. 2018 May 10;13(5):e0197232. doi: 10.1371/journal.pone.0197232 (PMC5944983; doi:10.1371/journal.pone.0197232)
Supplement: S2 Table — (DOCX) [file pone.0197232.s022.docx]

|  |  |  |  |
| --- | --- | --- | --- |

**Table S2. Plasmids expressing fragments of M.MpeI and M.SssI.**

| **plasmid** | **fragment** | **PCR template^1^** | **PCR primers^1^** |
| --- | --- | --- | --- |
| pB-Mpe[1-61] | Mpe[1-61] | pET28MpeI | AK418, AK463 |
| pOB-Mpe[62-395] | Mpe[62-395] | pET28MpeI | AK417, AK464 |
| pB-Mpe[361-244] | Mpe[361-244] | pB-tdM.MpeI | AK394, AK425 |
| pOB-Mpe[245-360] | Mpe[245-360] | pET28MpeI | AK393, AK426 |
| pB-Mpe[192-61] | Mpe[192-61] | pB-tdM.MpeI | AK418, AK442 |
| pOB-Mpe[62-191] | Mpe[62-191] | pET28MpeI | AK417, AK443 |
| pB-Mpe[280-61] | Mpe[280-61] | pB-tdM.MpeI | AK395, AK418 |
| pOB-Mpe[62-279] | Mpe[62-279] | pET28MpeI | AK396, AK417 |
| pB-Sss[1-57] | Sss[1-57] | pBNH-MSssI | AK413, AK449 |
| pOB-Sss[58-386] | Sss[58-386] | pBNH-MSssI | AK448, AK470 |
| pB-Sss[357-242] | Sss[357-242] | pB-tdM.SssI | AK429, AK446 |
| pOB-Sss[243-356] | Sss[243-356] | pBNH-M.SssI | AK428, AK447 |
| pOB-Sss[276-57] | Sss[276-57] | pB-tdM.SssI | AK449, AK468 |
| pB-Sss[58-275] | Sss[58-275] | pBNH-MSssI | AK448, AK467 |
| pB-Sss[58-242] | Sss[58-242] | pBNH-MSssI | AK448, AK429 |
| pOB-Sss[243-57] | Sss[243-57] | pB-tdM.SssI | AK428, AK449 |

1. Plasmids with names starting with pB- are based on pBAD24 (Ap^R^). Plasmids carrying names starting with pOB- are based on pOK-BAD (Kn^R^).
2. ^1^Template and primers used to PCR-amplify the respective gene segment.
